# Comparison of Glucose Lowering Effect of Metformin and Acarbose in Type 2 Diabetes Mellitus: A Meta-Analysis

**DOI:** 10.1371/journal.pone.0126704

**Published:** 2015-05-11

**Authors:** Shuyan Gu, Jihao Shi, Zhiliu Tang, Monika Sawhney, Huimei Hu, Lizheng Shi, Vivian Fonseca, Hengjin Dong

**Affiliations:** 1 Center for Health Policy Studies, School of Public Health, Zhejiang University School of Medicine, Hangzhou City, Zhejiang Province, China; 2 Sir Run Run Shaw Hospital, Zhejiang University School of Medicine, Hangzhou City, Zhejiang Province, China; 3 Health Economics and Outcome Research (HEOR), Bristol-Myers Squibb, Shanghai City, China; 4 College of Health Professions, Marshall University, Huntington, West Virginia, United States of America; 5 Department of Global Health Systems and Development, School of Public Health and Tropical Medicine, Tulane University, New Orleans, Louisiana, United States of America; 6 Section of Endocrinology, Department of Medicine, School of Medicine, Tulane University, New Orleans, Louisiana, United States of America; University of Florida, UNITED STATES

## Abstract

**Background:**

Metformin is the first-line oral hypoglycemic agent for type 2 diabetes mellitus recommended by international guidelines. However, little information exists comparing it with acarbose which is also commonly used in China. This study expanded knowledge by combining direct and indirect evidence to ascertain the glucose lowering effects of both drugs.

**Methods:**

PubMed (1980- December 2013) and China National Knowledge Infrastructure databases (1994-January 2014) were systematically searched for eligible randomized controlled trials from Chinese and English literatures. Meta-analysis was conducted to estimate the glucose lowering effects of metformin vs. acarbose, or either of them vs. common comparators (placebo or sulphonylureas), using random- and fixed-effect models. Bucher method with indirect treatment comparison calculator was applied to convert the summary estimates from the meta-analyses into weighted-mean-difference (WMD) and 95% confidence intervals (CIs) to represent the comparative efficacy between metformin and acarbose.

**Results:**

A total of 75 studies were included in the analysis. In direct comparison (8 trials), metformin reduced glycosylated hemoglobin (HbA_1c_) by 0.06% more than acarbose, with no significant difference (WMD,-0.06%; 95% CI, -0.32% to 0.20%). In indirect comparisons (67 trials), by using placebo and sulphonylureas as common comparators, metformin achieved significant HbA_1c_ reduction than acarbose, by -0.38% (WMD,-0.38%, 95% CI, -0.736% to -0.024%) and -0.34% (WMD, -0.34%, 95% CI, -0.651% to -0.029%) respectively.

**Conclusion:**

The glucose lowering effects of metformin monotherapy and acarbose monotherapy are the same by direct comparison, while metformin is a little better by indirect comparison. This implies that the effect of metformin is at least as good as acarbose's.

## Introduction

Type 2 diabetes mellitus (T2DM) is a chronic progressive metabolic disease and is reaching epidemic proportions in China. In adults 18 years and older in China, the prevalence of diabetes was 11.6%, with a total number of 113.9 million in 2010; China has become the country with the largest number of diabetic population [1]. T2DM accounts for at least 90% of all cases of diabetes [2]. This disease has brought great burden in terms of health care cost and socioeconomic consequences. Glycosylated hemoglobin (HbA_1c_) is the gold standard that reflects the glycemic control level, and Chinese Diabetes Society's (CDS) and American Diabetes Association’s (ADA) guidelines are taking HbA_1c_ < 7.0% as the glycemic control goal criteria [3, 4]. However, Chinese goal-achieving rate is poor, approximately only 39.7% of diabetics are with adequate glycemic control in 2010 [1].

The treatment of T2DM includes persistent lifestyle interventions, medical care, and patients’ self-management and education in order to prevent occurrence of acute diabetic complications and reduce risk of chronic complications. When lifestyle interventions can no longer achieve the HbA_1c_ goal, CDS, ADA, European Association for Study of Diabetes (EASD) and many other authoritative clinical practice guidelines recommend metformin as the first-line drug in either monotherapy or combination therapy [3, 4]. Furthermore, China has taken acarbose as one of the second-line drugs in treatment of diabetes [3].

As a selective hepatic insulin-sensitizing drug, metformin can reduce HbA_1c_ by 1.0%- 1.5% [3], by improving insulin sensitivity and decreasing intestinal absorption of glucose. It can either keep weight stability or reduce weight modestly for T2DM patients [3, 5]. Moreover, metformin has demonstrated long-term effectiveness and safety as medication for diabetes prevention [6]. Acarbose is an α-glucosidase inhibitor that inhibits the digestion and absorption of carbohydrates in small intestine, thus reducing the increase in blood-glucose concentrations after a carbohydrate load. It can reduce HbA_1c_ level by 0.5%, and is recommended for treating T2DM patients with high level of carbohydrate intake in China [3].

Due to the differences in mechanism and site of action between metformin and acarbose, they may have differences in glucose lowering effects. However, in use of oral hypoglycemic agents in China, both metformin (53.7%) and α-glucosidase inhibitors (including acarbose, 35.9%) are commonly accepted and widely used either as monotherapy or in combination with other oral agents or insulin for the treatment of T2DM [7]. It is also found that head-to-head direct comparison studies of both drugs are not many, while it is more common for them to compare with placebo or sulphonylureas. Therefore, we decided to directly (using meta-analysis) and indirectly (using indirect treatment comparison method) compare the results of the two drugs in reducing HbA_1c_ in order to provide a comprehensive picture of their differences in glucose lowering effects by systematically analyzing the English and Chinese literature.

## Materials and Methods

### Data sources and searches

Database searches were conducted to identify relevant studies comparing the glucose lowering effects of metformin and acarbose (head-to-head between them as direct comparison), or either of them with placebo or sulphonylureas (indirect comparisons) in patients with T2DM. PubMed (1980- December 2013) was searched to identify relevant English studies (S1 Table). Chinese studies were retrieved from China National Knowledge Infrastructure (CNKI) database (1994-January 2014). The following terms were used alone or in combination in the search: metformin, acarbose, type 2 diabetes mellitus, glibenclamide, glipizide, glimepiride, gliclazide and placebo. The references and citations of the retrieved studies and relevant reviews were also manually examined to identify additional studies.

### Study selection

We included randomized controlled trials (RCTs) evaluating the glucose lowering effects of metformin or acarbose for T2DM (18 years or older). The studies that compared a trial group receiving any daily doses of metformin or acarbose monotherapy with a control group receiving placebo or sulphonylureas monotherapy were selected, regardless of diet and/or physical exercise were used. Moreover, the included studies should take HbA_1c_ as the primary outcome indicator, with a follow-up duration of > 8 weeks. We excluded literature reviews, cross sectional studies, studies on animals or cell lines, and studies of type 1 diabetes or gestational diabetes.

Two reviewers independently screened titles and abstracts of the studies retrieved, and examined each potentially eligible study by reading full texts. Any disagreements were resolved with consultation of a third reviewer.

### Data extraction and quality assessment

A data extraction form was developed to record study characteristics, specific interventions and main results. Study characteristics include the key author’s name, publication year, study location, sample size, patient’s baseline information and study design. Intervention characteristics include daily dosage and route of medications, basic treatment and treatment duration. Main results include means and standard deviations (SDs) or standard errors (SEs) of HbA_1c_ levels.

We assessed the quality of the studies based on the following criteria: minimization of selection bias (randomization procedure, allocation concealment), minimization of performance bias (use of blinding), and minimization of attrition bias (dropout/loss to follow-up) [8–10].

- Randomization procedure: A = adequate (e.g., tables of random numbers, computer generated schemes, coin tossing); B = inadequate or unknown (e.g., odd or even date of birth, some rule based on date of admission, incompletely described).
- Allocation concealment: A = adequate (e.g., central allocation, sequentially numbered, opaque, sealed envelopes); B = inadequate or unknown (e.g., an open random allocation schedule, alternation or rotation, incompletely described).
- Use of blinding: A = adequate (e.g., blinding of participants and key study personnel ensured, and unlikely that the blinding could have been broken); B = mentioning of blinding but exact method unclear; C = non-blinded, inadequate or unknown.
- Dropout/loss to follow-up: A = overall dropout rate < 15%; B = overall dropout rate > 15%, or unknown.

The overall quality of each study was broadly subdivided into three categories: A = low risk of bias, high-quality trials; B = moderate risk of bias, moderate-quality trials; or C = high risk of bias, low-quality trials [8–10].

Two reviewers independently extracted and assessed each study, and any disagreements between the reviewers were resolved with consultation of a third reviewer.

### Data synthesis and analysis

#### Data synthesis

The studies were classified into two groups. One was the head-to-head comparison group, which included studies directly comparing the treatment effect of metformin and acarbose; the other was indirect comparison group, which included studies directly comparing the treatment effect of metformin with placebo or sulphonylureas and acarbose with placebo or sulphonylureas. We used placebo or sulphonylureas as a common comparator to perform indirect comparison of metformin and acarbose. Since HbA_1c_ is a continuous variable expressed using the same unit in all studies, we used the weighted mean difference (WMD) and 95% confidence intervals (CI) as summary statistic. The measure of the treatment effect is the changes in HbA_1c_ between baseline and endpoint values of the studies. Where the mean and SD of the change from baseline to endpoint were not reported in the original articles, the following equations were used to calculate them.

$${\text{Mean}}_{change}={\text{ Mean}}_{endpoint}–{\text{ Mean}}_{baseline};$$

$SD_{change}=\sqrt{{\left(SD_{baseline}\right)}^{2}+{\left(SD_{endpoint}\right)}^{2}-2\times r\times SD_{baseline}\times SD_{endpoint}}$, where *r* represents the correlation coefficient. We took r = 0.4 as a conservative estimate in this study [9, 10].

#### Statistical analysis

In the head-to-head (direct) comparison, we conducted a meta-analysis to estimate the glucose lowering effect of metformin versus acarbose. For the indirect comparison, in order to perform the adjusted indirect comparison for each of the outcomes of interest, four independent meta-analyses were performed for metformin or acarbose versus placebo or sulphonylureas as a common comparator (that is, metformin versus placebo, acarbose versus placebo, metformin versus sulphonylureas, and acarbose versus sulphonylureas, respectively). We also made subgroup analyses based on the daily drug doses, and Eastern and Western groups, in order to investigate the effects of dose levels on reduction in HbA_1c_ and the interaction of ethnicity and drug effects. Eastern refers to Asia and Western refers to Europe, Americas and Oceania, according to the regions where the studies were conducted.

The meta-analyses were carried out using RevMan software (version 5.2; Cochrane Collaboration). Heterogeneity was quantified by statistic I^2^. A fixed-effect model was used when no significant heterogeneity was detected among studies (P>0.10, I^2^ ≤50%), otherwise, a random-effect model was used. The Bucher-adjusted method with the indirect treatment comparison (ITC) calculator developed by Canadian Agency for Drugs and Technologies in Health (CADTH) was applied to convert the summary estimates from the meta-analyses of direct comparisons into WMD and 95% CIs to represent the comparative effect of metformin versus acarbose [11, 12]. The Bucher method of conducting indirect treatment comparisons in meta-analyses of RCTs had been commonly used and well validated [13].

#### Sensitivity analysis

Since studies in both direct and indirect comparisons might have differences in such aspects as quality, which might influence the result of meta-analysis, then, to confirm robustness of our findings, sensitivity analysis was conducted as the following three ways: changing effect model, removing the studies with higher risk of bias, or adjusting the drug doses.

## Results

**Fig 1:**
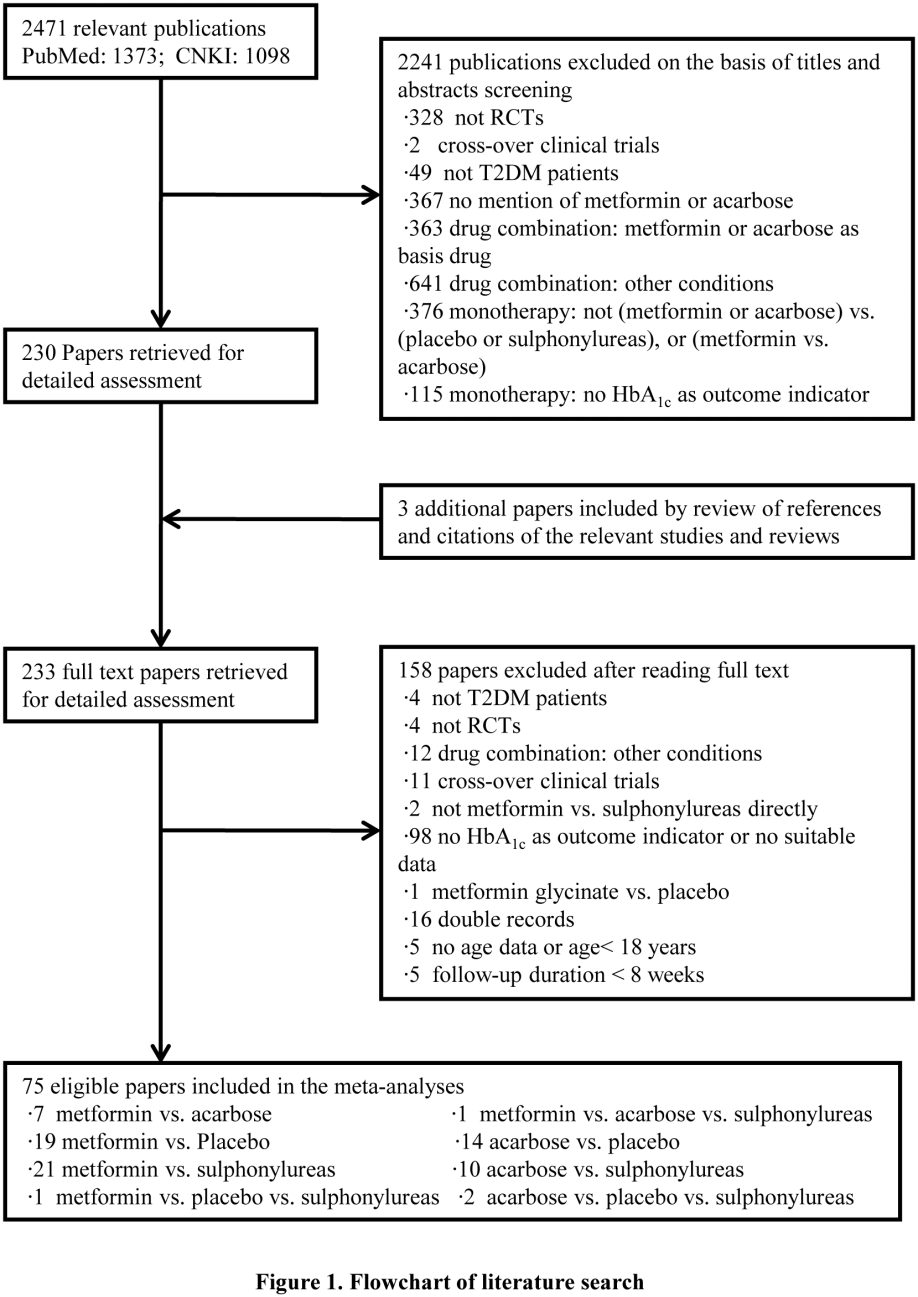
Flowchart of literature search. A detailed flow diagram that depicts search and selection processes.

The initial search identified a total of 2,471 citations, 1,373 of which were in English, 1,098 were in Chinese. 230 articles were retrieved for further review after titles and abstracts screening. In addition, three more articles were included after the manual review of the references and citations of the relevant studies. At last, 75 articles were identified eligible for our meta-analyses after examination of full text articles (Fig 1).

### Study characteristics and quality

All the eligible English and Chinese studies included in the meta-analyses were RCTs of monotherapy. The sample sizes of these studies varied from 18 to 520, with a follow-up duration ranging from 8 to 156 weeks, and most studies provided sufficient data about basic patient characteristics, such as age, disease duration and body mass index (BMI) (Table 1 and S2 Table). For study quality, 8 of 75 studies were classified as high-quality (A), 33 as moderate-quality (B), and 34 as low-quality (C). Among them, only 8 studies contributed data for head-to-head comparison of metformin with acarbose, but all of them were classified as C [14–21]. While in indirect comparison, 20 studies reported data on metformin and placebo with 3 as A, 16 as B, and 1 as C [22–41]; 16 studies on acarbose and placebo with 2 as A, 10 as B, and 4 as C [42–57]; 23 studies on metformin and sulphonylureas with 3 as A, 7 as B, and 13 as C [14, 38, 58–78]; and 13 studies on acarbose and sulphonylureas with 1 as B and 12 as C [14, 45, 47, 79–88] (S3 Table).

**Table 1:** Characteristics of Studies included in the head-to-head comparison.

Study	Location	Metformin Group						Acarbose Group						Intervention characteristics	
		Size	Age (y)	DM Duration (mo/y)	BMI (kg/m^2^)	HbA_1c_ (%)	Drug dose (mg/d)	Size	Age (y)	DM Duration (mo/y)	BMI (kg/m^2^)	HbA_1c_ (%)	Drug dose (mg/d)	Basic treatment	Duration (wk)
**Chen 2004 [14]**	China	32	20–44	ND	25.31 (0.62)	9.37 (1.8)	1500	32	20–44	ND	25.19 (0.53)	9.82 (1.73)	150	Null	36
**Chou 2013 [15]**	China	58	43.7 (8.5)	8.7 (2.3) mo	27.7 (4.1)	11.1 (4.3)	Initial: 750; 4wk later: 1500	58	43.7 (8.5)	8.7 (2.3) mo	27.3 (2.9)	10.5 (3.6)	Initial: 150; 4wk later: 300	diet	26
**Hong 2011 [16]**	China	68	41–75	1–9 y	NR	8.3 (2.32)	1500	69	41–75	1–9 y	NR	8.7 (2.09)	150	diet	8
**Tang 2013 [17]**	China	45	56.5 (7.2)	6.1 (0.7) y	24.8 (2.1)	11.54 (1.25)	750	45	57.9 (7.6)	6.4 (0.8) y	26.2 (2.4)	11.32 (1.18)	150	diet+exercise	13
**Wang 2005 [18]**	China	42	20–58	NR	25.2 (0.59)	9.37 (1.79)	Initial: 500; Max: 2250	42	20–58	NR	25.18 (0.52)	9.83 (1.71)	Initial: 50; Max: 200	diet+exercise	156
**Yang 2009 [19]**	China	15	30–65	NR	NR	9.37 (1.36)	1500	15	30–65	NR	NR	8.57 (2.76)	150	diet+exercise	12
**Zhang 2011 [20]**	China	30	35–60	ND	26.96 (1.34)	8.35 (0.93)	1500	30	35–60	ND	26.18 (0.72)	8.12 (1.14)	150	diet+exercise	12
**Zhu 2011 [21]**	China	33	52 (10)	ND	NR	8.2 (1.1)	1500	32	52 (10)	ND	NR	8.3 (1.3)	150	diet	26

Data are expressed as n, median (minimum-maximum), mean (SD); Size, sample size; DM, type 2 diabetes; BMI, body mass index; HbA_1c_,glycosylated hemoglobin; y, year; mo, month; mo/y, month or year; wk, week; ND, newly diagnosed; NR, not reported; Null, with no basic treatment;

### Effect in glucose lowering by head-to-head comparison

**Fig 2:**
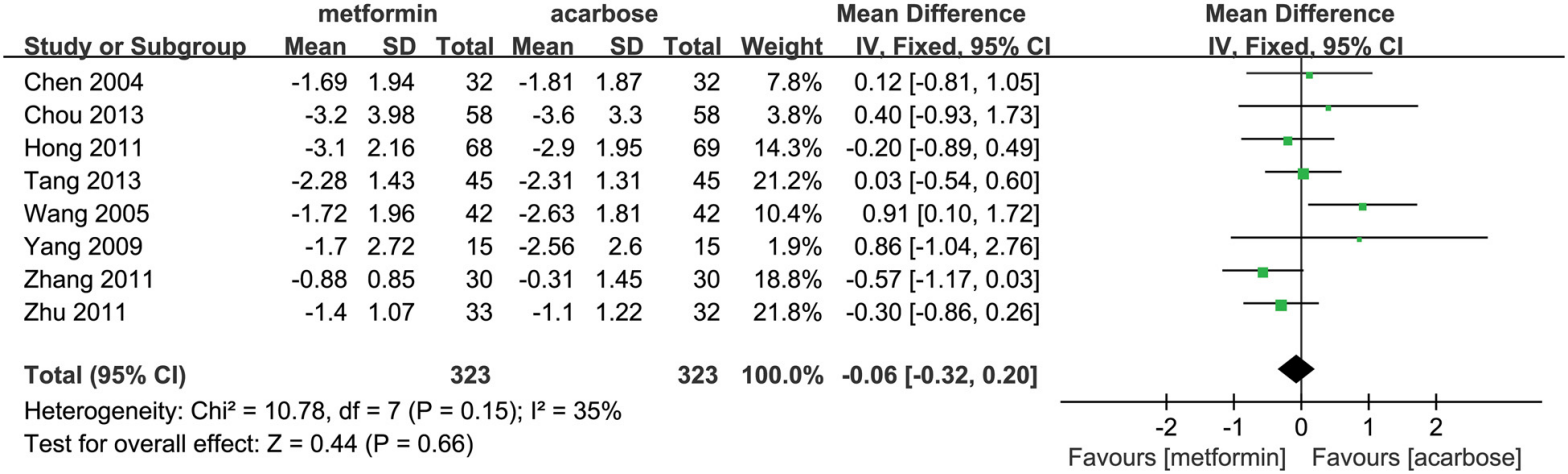
Glucose lowering effect (reduction of HbA_1c_) of metformin versus acarbose. The forest plot attained from the meta-analyses which provide the detailed data of difference in glucose lowering effect (reduction of HbA_1c_).

Eight studies were included in the meta-analysis for the comparison of metformin (323 patients) with acarbose (323 patients), all of which were Chinese studies [14–21]. Since the studies had homogeneity (*P* = 0.15, *I* ^2^ = 35%), we used a fixed-effect model for the meta-analysis. The result (WMD, -0.06%; 95% CI, -0.32% to 0.20%; *P* = 0.66; Fig 2) indicated that metformin reduced HbA_1c_ levels by 0.06% more than acarbose, but this difference was not significant. Results from sensitivity analysis by using a random-effect model confirmed the findings that the effects of both drugs had no significant difference (WMD, -0.01%; 95% CI, -0.35% to 0.33%; *P* = 0.96; S1 Fig).

### Effect in glucose lowering by indirect comparisons (using placebo as comparator)

#### Metformin versus Placebo

**Fig 3:**
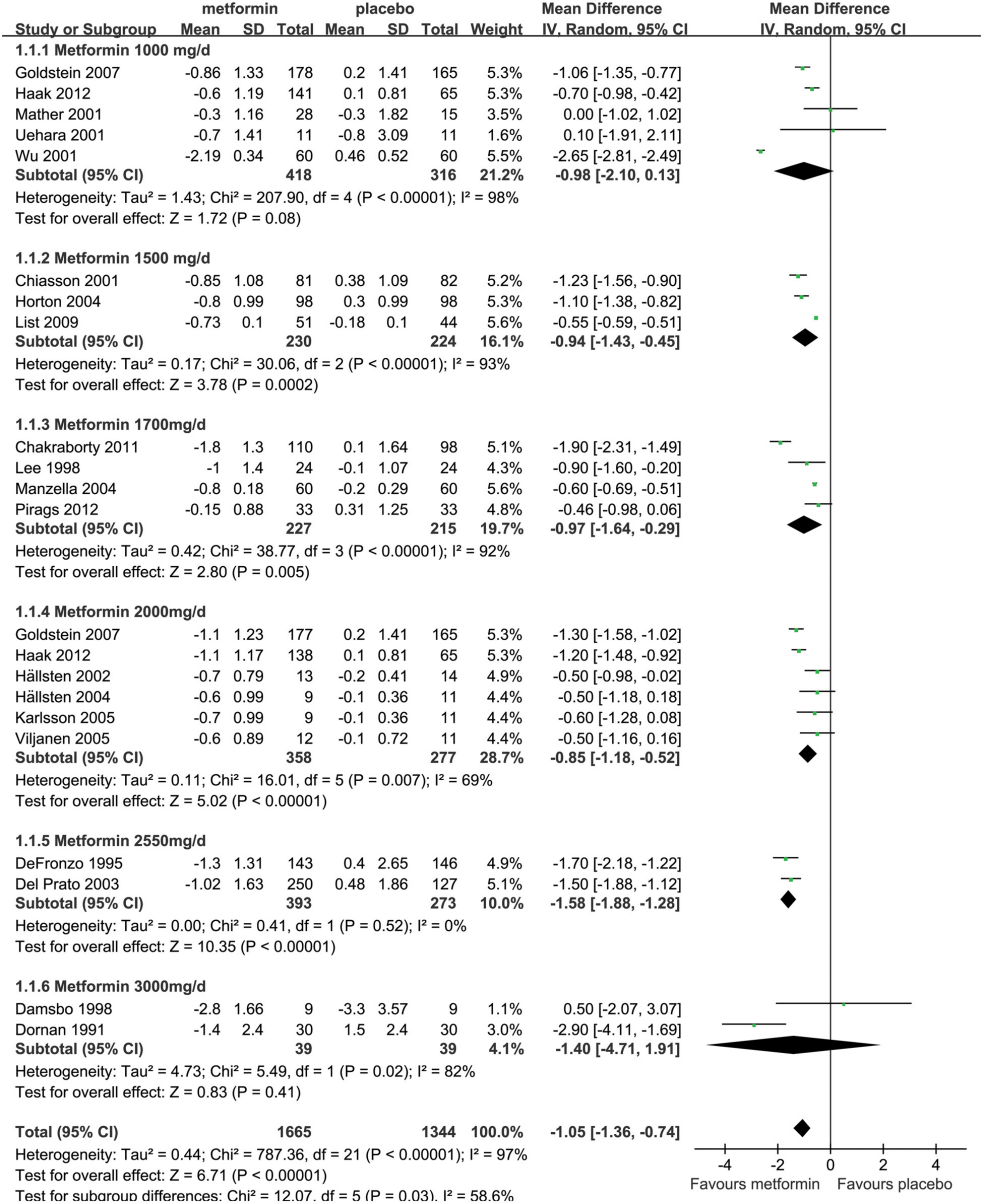
Glucose lowering effect (reduction of HbA_1c_) of metformin versus placebo. The forest plot attained from the meta-analyses which provide the detailed data of difference in glucose lowering effect (reduction of HbA_1c_).

Metformin was compared with placebo in 20 studies. The pooled sample of metformin group and placebo group were 1,665 and 1,344, respectively [22–41]. Meta-analysis of the entire group used a random-effect model due to the substantial heterogeneity among studies (*P*<0.001, *I* ^2^ = 97%). The result revealed that, compared with placebo, metformin performed significantly better (by 1.05%) in reducing HbA_1c_ levels (WMD,-1.05%; 95% CI, -1.36% to -0.74%; *P*<0.001; Fig 3).

Metformin reduced HbA_1c_ levels by 0.98% and 1.40% more than placebo in studies using doses of 1000mg/d (WMD,-0.98%, 95%CI, -2.10% to 0.13%; *P* = 0.08) and 3000mg/d (WMD, -1.40%, 95%CI, -4.71% to 1.91%; *P* = 0.41), respectively, but both differences were not significant. Whereas in studies using doses of 1500mg/d, 1700mg/d, 2000mg/d and 2550mg/d, the HbA_1c_ reductions were 0.94% (WMD, -0.94%, 95%CI, -1.43% to -0.45%; *P*<0.001), 0.97% (WMD, -0.97%, 95%CI, -1.64% to -0.29%; *P* = 0.005), 0.85% (WMD, -0.85%, 95%CI, -1.18% to -0.52%; *P*<0.001), and 1.58% (WMD, -1.58%, 95%CI, -1.88% to -1.28%; *P*<0.001), respectively, and all the differences were significant (Fig 3). Besides, metformin in both Western (WMD, -0.92%, 95%CI, -1.09% to -0.75%; *P*<0.001) and Eastern (WMD, -2.30%, 95%CI, -3.03% to -1.57%; *P*<0.001) subgroups also showed significant HbA_1c_ lowering effects compared to placebo, whilst metformin appeared to perform better in Eastern group (S2 Fig). The results indicated that metformin could effectively reduce HbA_1c_ levels for T2DM patients.

Sensitivity analyses were performed by using a fixed-effect model and removing one low-quality study [34]. The results showed that metformin achieved 0.72% (WMD, -0.72%, 95%CI, -0.75% to -0.69%; *P*<0.001; S3 Fig) and 1.08% (WMD, -1.08%, 95%CI, -1.40% to -0.76%; *P*<0.001; S4 Fig) greater HbA_1c_ reductions than placebo, respectively, which further showed that metformin had significant HbA_1c_ lowering effect for T2DM patients, and the result was robust.

#### Acarbose versus Placebo

**Fig 4:**
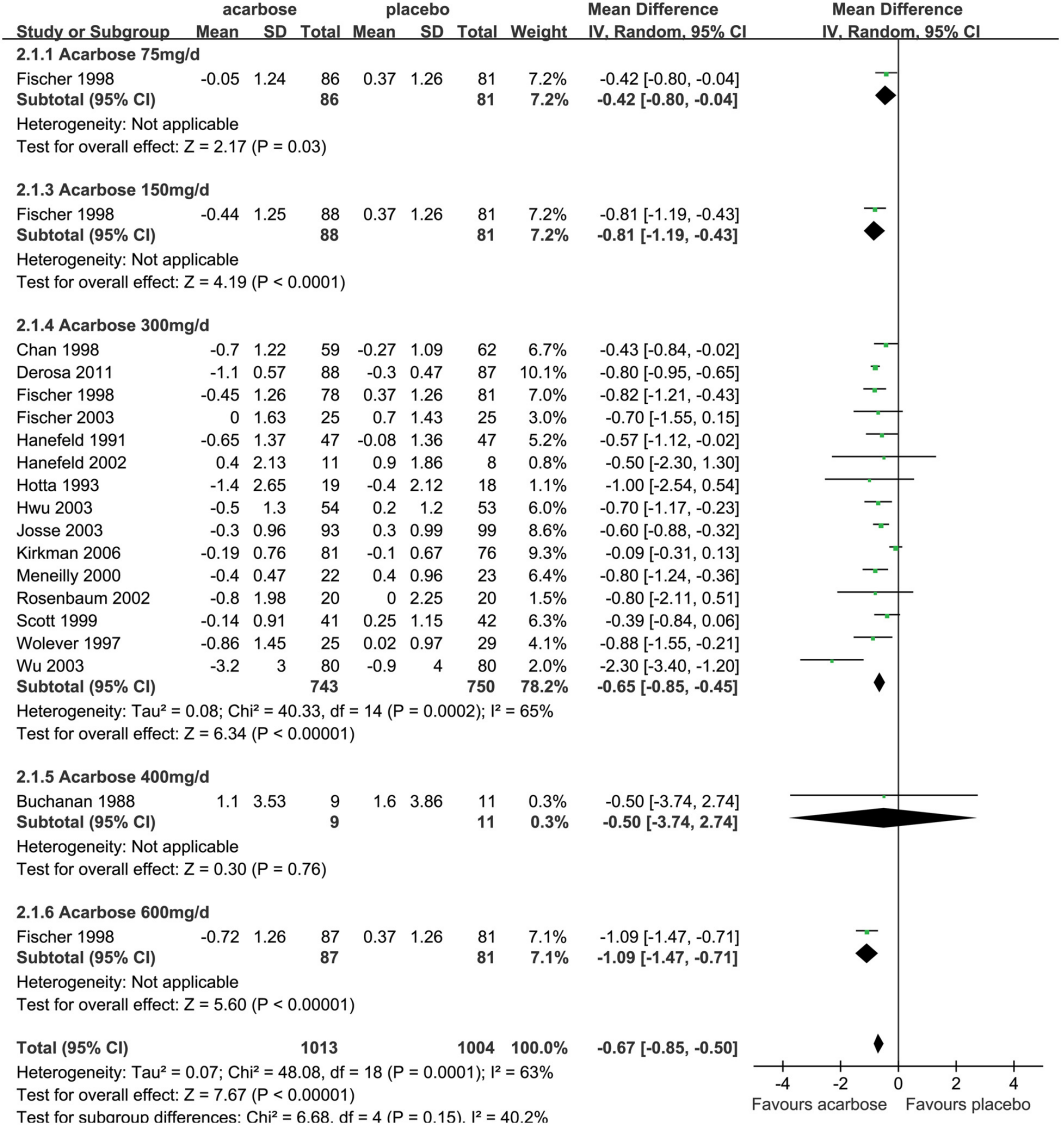
Glucose lowering effect (reduction of HbA_1c_) of acarbose versus placebo. The forest plot attained from the meta-analyses which provide the detailed data of difference in glucose lowering effect (reduction of HbA_1c_).

Acarbose was compared with placebo in 16 studies, with the pooled sample of 1,013 acarbose patients and 1,004 placebo patients [42–57]. Meta-analysis of the entire group using a random-effect model (*P*<0.001, *I* ^2^ = 63%) indicated that acarbose achieved a 0.67% greater reduction in HbA_1c_ levels compared to placebo (WMD, -0.67%, 95%CI, -0.85% to -0.50%; *P*<0.001; Fig 4), and the difference was significant.

In the dosage subgroups using 75mg/d, 150mg/d, 300mg/d, and 600mg/d doses of acarbose, the reductions in HbA_1c_ levels were 0.42% (WMD, -0.42%, 95%CI, -0.80% to -0.04%; *P* = 0.03), 0.81% (WMD, -0.81%, 95%CI, -1.19% to -0.43%; *P*<0.001), 0.65% (WMD, -0.65%, 95%CI, -0.85% to -0.45%; *P*<0.001), and 1.09% (WMD, -1.09%, 95%CI, -1.47% to -0.71%; *P*<0.001) more than placebo, respectively, and all the differences were significant (Fig 4). In Western and Eastern subgroups, acarbose was also found to significantly reduce HbA_1c_ levels compared to placebo, while it might perform better in Eastern (WMD, -0.94%, 95%CI, -1.59% to -0.30%; *P* = 0.004) than Western (WMD, -0.65%, 95%CI, -0.83% to -0.46%; *P*<0.001) group (S5 Fig). The results revealed that acarbose could effectively reduce HbA_1c_ levels for T2DM patients.

Sensitivity analyses were conducted by using a fixed-effect model and removing four low-quality studies [45, 47, 52, 53]. The results showed that acarbose can significantly reduce HbA_1c_ level by 0.64% (WMD, -0.64%, 95%CI, -0.72% to -0.55%; *P*<0.001; S6 Fig) and 0.67% (WMD, -0.67%, 95%CI, -0.86% to -0.47%; *P*<0.001; S7 Fig) more than placebo, respectively, which confirmed that acarbose had significant HbA_1c_ lowering effect for T2DM patients, and the result was robust.

#### Metformin versus Acarbose

In order to observe the differences between metformin and acarbose in glucose lowering effect more intuitively, an indirect treatment comparison (ITC) of metformin with acarbose was conducted using placebo as the common comparator. The results showed statistically significant differences, that metformin reduced HbA_1c_ levels by 0.38% more than acarbose (WMD, -0.38%, 95%CI, -0.736% to -0.024%; Table 2). Thus, the indirect comparison suggested that glucose lowering effect of metformin was superior to that of acarbose.

**Table 2:** Glucose lowering effect (reduction of HbA_1c_) of Metformin versus Acarbose.

Comparisons Type	Group	Results [Mean Difference (95%CI) (%)]	*P* values
**Direct Comparison**	Metformin vs. Acarbose	-0.06 [-0.32, 0.20] ^a^	0.66
**Indirect Comparison (using Placebo as comparator)**	Metformin vs. Placebo	-1.05 [-1.36, -0.74] ^a^	<0.001
	Acarbose vs. Placebo	-0.67 [-0.85, -0.50] ^a^	<0.001
	Metformin vs. Acarbose	-0.38 [-0.736,-0.024] ^b^	—^d^
**Indirect Comparison (using Sulphonylureas as comparator)**	Metformin vs. Sulphonylureas	-0.18 [-0.39, 0.04] ^a^	0.11
	Acarbose vs. Sulphonylureas	0.16 [-0.07, 0.38] ^a^	0.17
	Metformin vs. Acarbose	-0.34 [-0.651,-0.029] ^c^	—^d^

**Fig 5:**
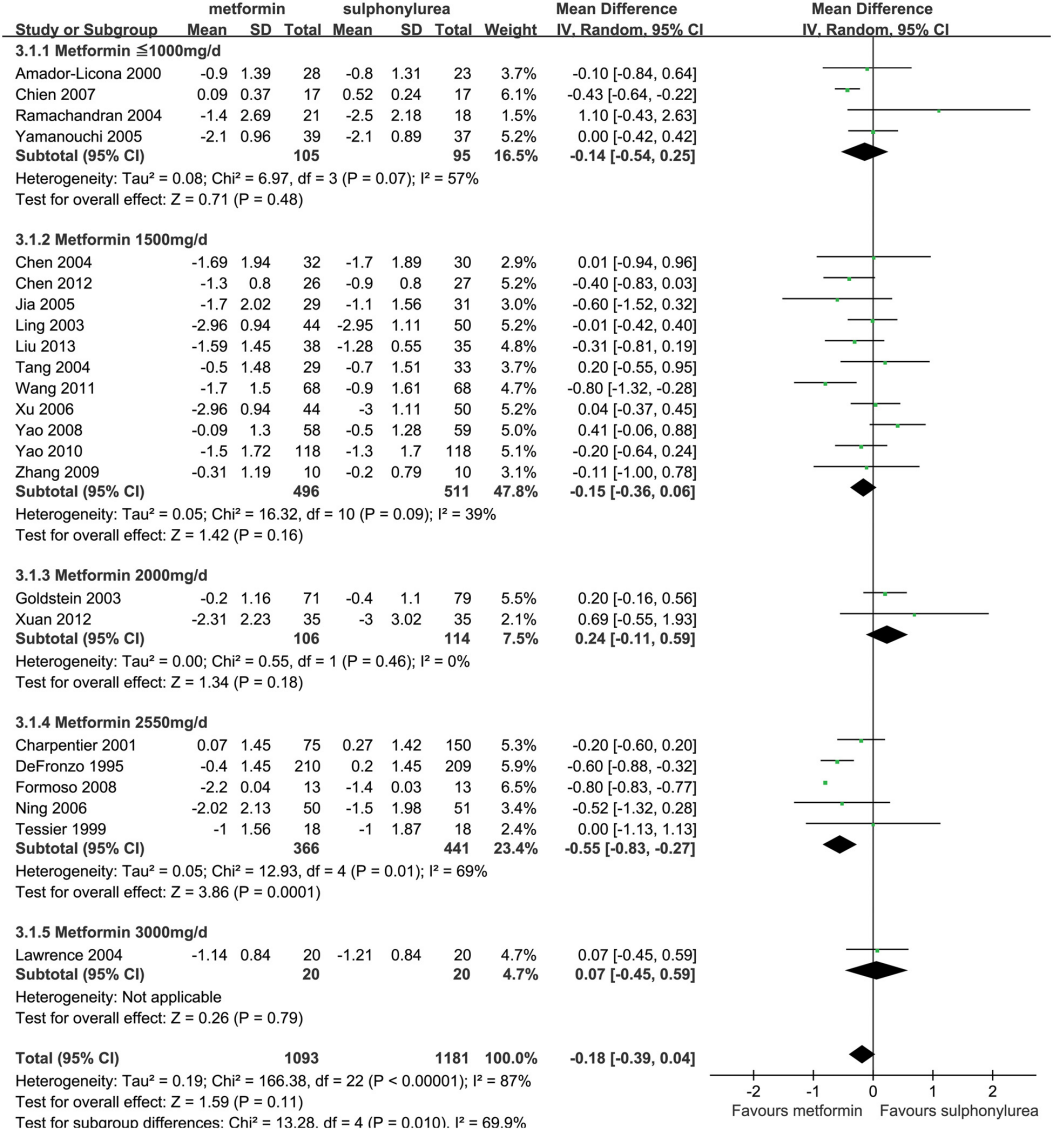
Glucose lowering effect (reduction of HbA_1c_) of metformin versus sulphonylureas. The forest plot attained from the meta-analyses which provide the detailed data of difference in glucose lowering effect (reduction of HbA_1c_).

**Fig 6:**
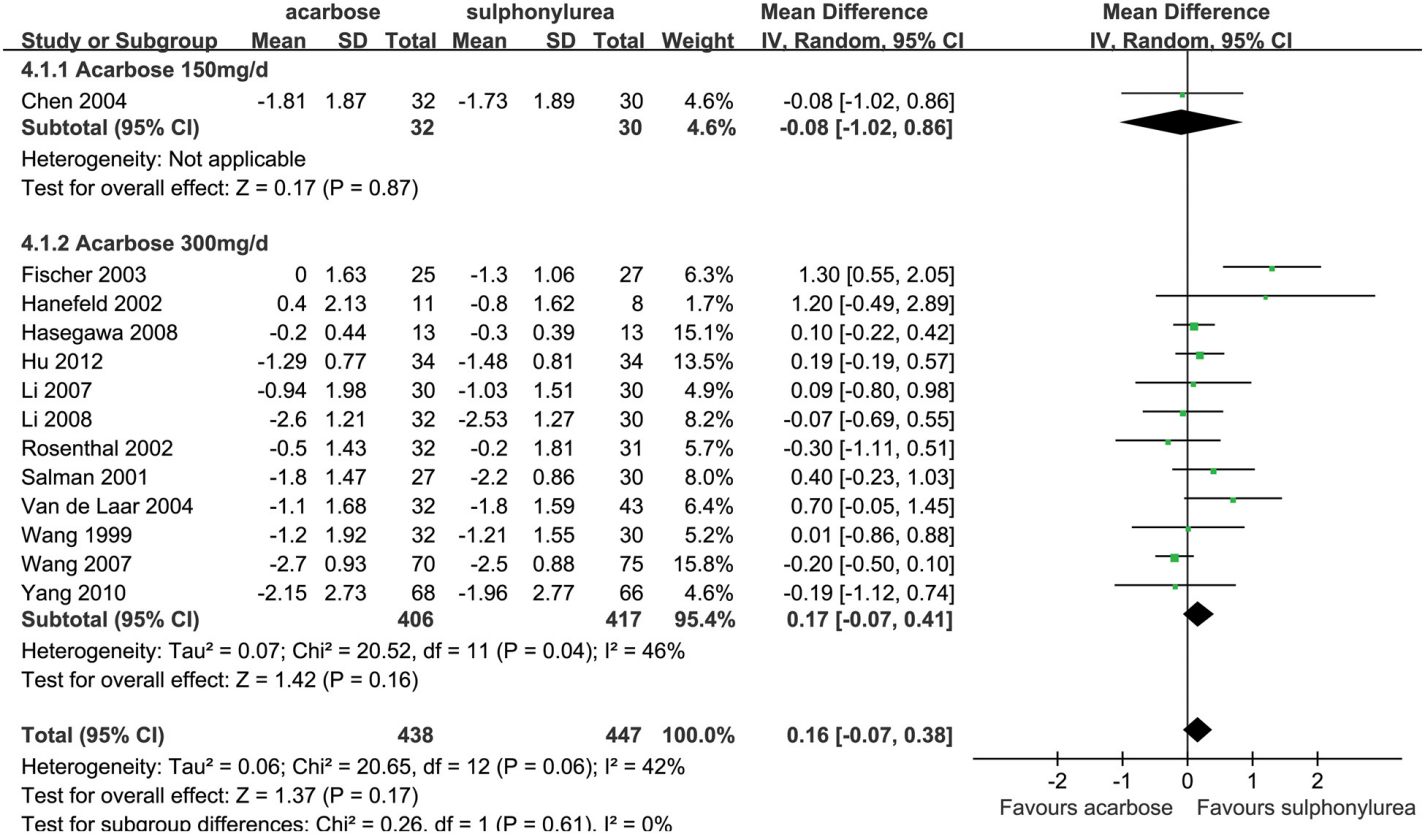
Glucose lowering effect (reduction of HbA_1c_) of acarbose versus sulphonylureas. The forest plot attained from the meta-analyses which provide the detailed data of difference in glucose lowering effect (reduction of HbA_1c_).

This table summarized the overall meta-analysis results of Figs 2, 3, 4, 5 and 6, as well as the converted indirect comparison results by using placebo or sulphonylureas as common comparator, which may objectively present the glucose lowering effect of metformin vs. acarbose from both direct and indirect perspectives.

^a^ The summary results of meta-analysis.

^b^ By using placebo as the common comparator, we applied the Bucher-adjusted method with the indirect treatment comparison calculator to convert the summary estimates form the meta-analyses of metformin vs. placebo and acarbose vs. placebo into WMD and 95% CIs to represent the comparative effect of metformin vs. acarbose.

^c^ By using sulphonylureas as the common comparator, we applied the Bucher-adjusted method with the indirect treatment comparison calculator to convert the summary estimates form the meta-analyses of metformin vs. sulphonylureas and acarbose vs. sulphonylureas into WMD and 95% CIs to represent the comparative effect of metformin vs. acarbose.

^d^ We cannot get P values because in the Indirect Treatment Comparison Calculator, we have to make indirect treatment comparisons in the "Main Window" (not in the "Requested Weights Window") due to the number of studies larger than pre-designed 20.

### Effect in glucose lowering by indirect comparisons (using Sulphonylureas as comparator)

#### Metformin versus Sulphonylureas

A total of 23 studies were included in the meta-analysis for the comparison of metformin (1,093 patients) with sulphonylureas (1,181 patients) [14, 38, 58–78]. Owing to the heterogeneity between the studies, we used a random-effect model (*P*<0.001, *I* ^2^ = 87%) in meta-analysis of the entire group. Result showed that metformin did not significant differ in HbA_1c_ reduction (WMD, -0.18%, 95%CI, -0.39% to 0.04%; *P* = 0.11; Fig 5) compared to sulphonylureas.

In dosage subgroup analysis, metformin reduced HbA_1c_ levels by 0.55% (WMD, -0.55%, 95%CI, -0.83% to -0.27%; *P*<0.001) more than sulphonylureas in studies using doses of 2550mg/d. Whereas in studies using doses of 2000mg/d or lower doses (< = 1000 mg or 1500 mg), the reduction of HbA_1c_ by metformin was similar to that by sulphonylureas (Fig 5). In Western and Eastern subgroups, both Western (WMD, -0.26%, 95%CI, -0.63% to 0.12%; *P* = 0.18) and Eastern (WMD, -0.16%, 95%CI, -0.34% to 0.02%; *P* = 0.09) subgroups indicated similar HbA_1c_ lowering effects of metformin and sulphonylureas (S8 Fig).

Sensitivity analyses were performed by removing several low-quality studies [14, 58, 60, 62, 64–66, 68, 73, 75–78] and adjusting drug dose (set ≥ 1500mg/d as maximum or maintenance dose of metformin). The results showed that metformin can reduce HbA_1c_ levels by 0.23% (WMD, -0.23%, 95%CI, -0.46% to 0.01%; *P* = 0.06; S9 Fig) and 0.19% (WMD, -0.19%, 95%CI, -0.44% to 0.06%; *P* = 0.13; S10 Fig) more than sulphonylureas, respectively, but the differences were not significant, which confirmed a result of comparable HbA_1c_ lowering effects between metformin and sulphonylureas.

#### Acarbose versus Sulphonylureas

A total of 13 studies were included in the meta-analysis for the comparison of acarbose (438 patients) with sulphonylureas (447 patients) [14, 45, 47, 79–88]. Meta-analysis of change in HbA_1c_ levels between these two drugs was performed using a random-effect model (*P* = 0.06, *I* ^2^ = 42%) in the entire group, the result revealed that acarbose reduced HbA_1c_ levels by 0.16% less than sulphonylureas (WMD, 0.16%, 95%CI, -0.07% to 0.38%; *P* = 0.17; Fig 6), but this difference was not significant.

The dosage subgroup analysis also highlighted a similar result (WMD, 0.17%, 95%CI, -0.07% to 0.41%; *P* = 0.16; Fig 6). In Western subgroup, acarbose reduced HbA_1c_ levels by 0.59% less than sulphonylureas (WMD, 0.59%, 95%CI, 0.03% to 1.15%; *P* = 0.04), while it was similar to sulphonylureas in Eastern subgroup (WMD, -0.01%, 95%CI, -0.18% to 0.16%; *P* = 0.91), which implies a potential better efficacy of acarbose in Eastern subgroup (S11 Fig).

Sensitivity analyses were conducted by using a fixed-effect model and adjusting drug doses (set ≥ 300 mg/d as maximum or maintenance dose of acarbose). The results showed that acarbose reduced HbA_1c_ level by 0.09% (WMD, 0.09%, 95%CI, -0.06% to 0.25%; *P* = 0.22; S12 Fig) and 0.17% (WMD, 0.17%, 95%CI, -0.07% to 0.41%; *P* = 0.16; S13 Fig) less than sulphonylureas, respectively, but the differences were not significant. Sensitivity analyses highlighted that acarbose was similar to sulphonylureas in HbA_1c_ lowering effect, and the results were robust.

#### Metformin versus Acarbose

We performed an ITC of metformin with acarbose by using sulphonylureas as the common comparator, which revealed that metformin achieved a statistically greater reduction of 0.34% in HbA_1c_ levels compared to acarbose (WMD, -0.34%, 95%CI, -0.651% to -0.029%; Table 2). Thus, the glucose lowering effect of metformin was superior to that of acarbose.

## Discussion

In the context of health technology assessment on medications, comparative evidence is essential to assist in the decision-making for formulary listing. A systematic review of RCTs that directly compared two comparative interventions would generally be regarded as the gold standard to support healthcare decisions. However, such direct RCT evidence is often limited or unavailable, thus has led to an increased use of indirect comparison methods to estimate the comparative efficacy of interventions [89, 90]. In this study, we find head-to-head (direct) comparison articles for evaluation of the treatment efficacy of metformin and acarbose are so far insufficient, therefore, the method of adjusted indirect comparison is further adopted to assess these two drugs.

Metformin and acarbose are two effective oral hypoglycemic agents which can effectively reduce the blood glucose for T2DM patients and widely used in China. However, due to the differences in mechanism and site of action between these two drugs, their glucose lowering effects may have some comparative differences. The head-to-head comparison shows that metformin performs similarly in reducing HbA_1c_ levels compared to acarbose. While in indirect comparisons, when placebo or sulphonylureas was served as a common comparator, metformin both shows a significantly greater reduction in HbA_1c_ levels. Metformin seems superior to acarbose in glycemic control for T2DM patients. Since metformin has additional mechanisms above and beyond reductions in intestinal glucose absorption, including improved peripheral insulin sensitivity and reduced hepatic glucose production, compared to acarbose [3], which may be able to account partly for greater HbA_1c_ reductions. However, acarbose may perform better in Eastern group than Western group, whereas metformin has no obvious difference. The reasons may be partly explained by differences in extrinsic factors (dietary patterns), intrinsic factors (genetics), an interaction of both factors as well as many other factors between different ethnicities [91]. Since acarbose has specific action mechanism of inhibiting digestion and absorption of carbohydrates in small intestine [3], it may perform better in patients consuming higher levels of carbohydrate in Eastern country [9, 92]. Meanwhile, there may be genetic diversity of α-glucosidase in different ethnic populations which may impact the biochemical effect of acarbose [91].

Indirect comparison of competing interventions can be generally defined as a comparison of different treatments for a clinical indication by using data from separate RCTs, in contrast to direct head-to-head RCTs [90]. Indirect comparison on the basis of a common comparator can maintain certain strengths of randomized allocation of patients for evaluating comparative efficacy of treatment [11]. The adjusted indirect comparison can partially take into account the baseline risk and other prognostic factors of participants in different trials, and incorporate more uncertainty into its result by providing a wider confidence interval [93]. When there is no or insufficient direct randomized evidence, it may provide useful or supplementary information in estimating relative efficacy of competing interventions [13, 93]. Biases and differences in methodology, outcome measurement, the populations or study settings and design in trials can inevitably impact the validity of adjusted indirect comparison. Besides, consistent (external validity) of relative efficacy of an intervention in participants across different trials is the key hypothesis to get indirect estimate be valid. However, external validity of trial results is often low attributable to atypical treatment and restricted inclusion criteria, exclusion of patients, and atypical clinical settings where trials were conducted [13, 94]. There are concerns that indirect comparison may be subject to greater bias than direct comparison and may overestimate the effect of interventions [11]. On the other hand, although direct randomised evidence is generally regarded to be the best, such evidence may not always be reliable attributable to several potential limitations in randomised trials including lack of concealment of allocation, unbalanced exclusions between groups after randomization, paucity of blinding of outcome measurement and publication bias [13]. Results of indirect comparison usually but not always agree with that of direct comparison. Inconsistency between the direct and indirect comparisons may be explained by fewer trials included in analyses, the extent of heterogeneity in meta-analyses, the play of chance, and bias in the direct or indirect comparison [90]. In this study, there were only 8 trials (646 patients) in direct comparison, which were rather smaller in sample size, compared to the 67 trials (8185 patients) in indirect comparisons. The number and sample size might explain the inconsistency in the results of direct and indirect comparisons, and demonstrate the reliability of the results drawn from indirect comparisons rather than direct comparison to a certain extent. Also, the quality of studies in direct comparison was poor, which might damage the credibility of the results of direct comparison compared to that of the indirect comparisons, so the potential great risk bias might also account for part of the inconsistency. Finally, the massive unexplainable heterogeneity in the meta-analyses of indirect comparisons was also associated with the mentioned inconsistency. The inherent limitations of indirect treatment comparison method might account for the unexplainable heterogeneity in indirect comparisons, and as well negatively affect the outcome of the study; however, the great sample size in indirect comparison might enhance its results’ reliability. In spite of the inconsistency, the results trend to show that metformin monotherapy generally had achieved slightly better glycemic control for T2DM compared to acarbose.

Nathan 2009 [5] and Inzucchi 2012 [95] had previously reported that metformin would lower HbA_1c_ levels by 1.0%-2.0%, and could keep either weight stability or modest weight loss without causing hypoglycemia. In spite of the tolerable gastrointestinal side effects, metformin was likely to reduce cardiovascular disease (CVD) events, which was demonstrated by the United Kingdom Prospective Diabetes Study (UKPDS) [96]. While α-glucosidase inhibitors (including acarbose) were less effective in lowering glycemia than metformin, for they reduced HbA_1c_ levels by 0.5%-0.8% [5]. The side effects of α-glucosidase inhibitors were also gastrointestinal adverse effects, which were similar to metformin. Though the advantages of α-glucosidase inhibitors were to primarily lower postprandial glucose levels, without causing hypoglycemia and malabsorption, they could not attain weight loss similar to metformin, and their effects on CVD outcomes remains unclear [5, 95]. Furthermore, international guidelines also affirmed the position of metformin in the treatment of T2DM, and recommended metformin as the first-line therapy for T2DM patients [3, 4]; while acarbose was recognized as one of the second-line therapies in China [3].

However, lack of large head-to-head direct comparison clinical trials, comparing the glucose lowering effect of metformin and acarbose in T2DM, made it difficult to find evidence to support the ideas of previous studies. The study of Zhou [9], which compared the glucose lowering effect of acarbose in T2DM consuming an eastern versus western diet, reported that acarbose reduced HbA_1c_ levels by 0.06% and 0.09% less than metformin in eastern and western diet group, respectively, both with no significant differences. Acarbose generally had a similar ability in glycemic control as metformin regardless of diet type. Our direct comparison result is consistent with Zhou's study, although the small sample sizes (588 patients) in Zhou’s study might impact the results’ reliability. The Cochrane review [97] suggested that α-glucosidase inhibitors failed to show more benefit for glycemic control, body weight, or lipids, than metformin, and metformin may be the first therapeutic option in T2DM with overweight or obesity. Our findings are roughly consistent with the previous studies in the aspect of glucose lowering effects of these two drugs, and our study has provided much needed evidence for the clinical medication choice in China where both metformin and acarbose are commonly used.

From methodological point of view, meta-analysis has some weakness, such as low quality of study, publication bias, massive unexplainable heterogeneity. In this study, we used sensitivity analysis to remove low-quality studies, systematically search literature published in English or Chinese to reduce the publication bias and random-effects model to account for the massive unexplainable heterogeneity. While methodological controversies of indirect comparison remained [13, 90, 98], however, we used Bucher method because it is widely recognized and well accepted as it preserves the power of randomization in original trials when calculating the magnitude of treatment effect [11]. The comparative drug efficacy generated by the indirect comparisons need to be interpreted with caution owing to study population heterogeneity and variation in study design [90]. Furthermore, in our study, the drug dosage was not exactly the same in all studies retrieved, which may result in the bias in the estimation of the effect size between the two drugs because different dosage may have different effect in reducing HbA_1c_ level. Considering the short duration of trials included in our analysis (8 to 156 weeks), the generalizability of our results may be limited attributable to paucity of long-term antibiabetic treatment evidence.

Despite these limitations, our study has noteworthy strengths in that it is the first systematic review and meta-analysis on the topic of comparison of metformin monotherapy with acarbose monotherapy in glycemic control for T2DM patients, with either direct or indirect evidence took into account. To our knowledge, this approach of using both methods is able to give a comprehensive summary for the differences between these two drugs in glucose lowering effect. We also expanded the knowledge by merging information from Chinese and English clinical studies for the first time, to fill the knowledge gap in this field.

In conclusion, the glucose lowering effects of metformin monotherapy and acarbose monotherapy are the same by direct comparison, while metformin monotherapy is a little better by indirect comparison. This means that the glucose lowering effects of metformin monotherapy is at least as good as acarbose monotherapy.

## Supporting Information

S1 PRISMA Checklist PRISMA 2009 Checklist. (DOC)

S1 Fig Sensitivity analysis: Glucose lowering effect (reduction of HbA_1c_) of metformin versus acarbose (use a random-effect model instead). (PDF)

S2 Fig Glucose lowering effect (reduction of HbA_1c_) of metformin versus placebo (Western and Eastern as subgroups). (PDF)

S3 Fig Sensitivity analysis: Glucose lowering effect (reduction of HbA_1c_) of metformin versus placebo (use a fixed-effect model instead). (PDF)

S4 Fig Sensitivity analysis: Glucose lowering effect (reduction of HbA_1c_) of metformin versus placebo (remove one low-quality study [34] from the original data set). (PDF)

S5 Fig Glucose lowering effect (reduction of HbA_1c_) of acarbose versus placebo (Western and Eastern as subgroups). (PDF)

S6 Fig Sensitivity analysis: Glucose lowering effect (reduction of HbA_1c_) of acarbose versus placebo (use a fixed-effect model instead). (PDF)

S7 Fig Sensitivity analysis: Glucose lowering effect (reduction of HbA_1c_) of acarbose versus placebo (remove four low-quality studies [45, 47, 52, 53] from the original data set). (PDF)

S8 Fig Glucose lowering effect (reduction of HbA_1c_) of metformin versus sulphonylurea (Western and Eastern as subgroups). (PDF)

S9 Fig Sensitivity analysis: Glucose lowering effect (reduction of HbA_1c_) of metformin versus sulphonylureas (remove thirteen low-quality studies [14, 58, 60, 62, 64–66, 68, 73, 75–78] from the original data set). (PDF)

S10 Fig Sensitivity analysis: Glucose lowering effect (reduction of HbA_1c_) of metformin versus sulphonylureas (adjust drug doses, set ≥ 1500mg/d as maximum or maintenance dose of metformin). (PDF)

S11 Fig Glucose lowering effect (reduction of HbA_1c_) of acarbose versus sulphonylurea (Western and Eastern as subgroups). (PDF)

S12 Fig Sensitivity analysis: Glucose lowering effect (reduction of HbA_1c_) of acarbose versus sulphonylureas (use a fixed-effect model instead). (PDF)

S13 Fig Sensitivity analysis: Glucose lowering effect (reduction of HbA_1c_) of acarbose versus sulphonylureas (adjust drug doses, set ≥ 300 mg/d as maximum or maintenance dose of acarbose). (PDF)

S1 Table Search strategy. (DOC)

S2 Table Characteristics of Studies included in the indirect comparisons. (DOC)

S3 Table Quality of Studies included in the meta-analyses. (DOC)
