# Supplementary material for: Comparison of Glucose Lowering Effect of Metformin and Acarbose in Type 2 Diabetes Mellitus: A Meta-Analysis
Source: PLoS One. 2015 May 11;10(5):e0126704. doi: 10.1371/journal.pone.0126704 (PMC4427275; doi:10.1371/journal.pone.0126704)
Supplement: S1 Fig — (PDF) [file pone.0126704.s002.pdf]

	metformin			acarbose				Mean Difference
Study or Subgroup	Mean	SD	Total	Mean	SD	Total	Weight	IV, Random, 95% CI
Zhu 2011	-1.4	1.07	33	-1.1	1.22	32	18.8%	-0.30 [-0.86, 0.26]
Zhang 2011	-0.88	0.85	30	-0.31	1.45	30	17.4%	-0.57 [-1.17, 0.03]
Yang 2009	-1.7	2.72	15	-2.56	2.6	15	3.0%	0.86 [-1.04, 2.76]
Wang 2005	-1.72	1.96	42	-2.63	1.81	42	12.1%	0.91 [0.10, 1.72]
Tang 2013	-2.28	1.43	45	-2.31	1.31	45	18.5%	0.03 [-0.54, 0.60]
Hong 2011	-3.1	2.16	68	-2.9	1.95	69	14.8%	-0.20 [-0.89, 0.49]
Chou 2013	-3.2	3.98	58	-3.6	3.3	58	5.6%	0.40 [-0.93, 1.73]
Chen 2004	-1.69	1.94	32	-1.81	1.87	32	9.9%	0.12 [-0.81, 1.05]
**Total (95% CI)**			**323**			**323**	**100.0%**	**-0.01 [-0.35, 0.33]**

Heterogeneity: $Tau^2 = 0.08$; $Chi^2 = 10.78$, df = 7 (P = 0.15); $I^2 = 35\%$

Test for overall effect: Z = 0.05 (P = 0.96)

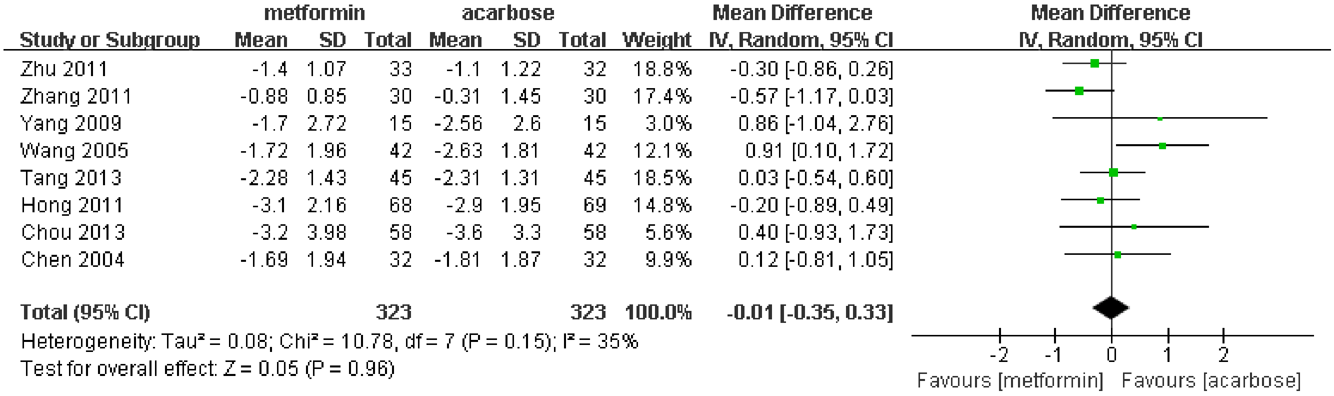

S1 Fig. Sensitivity analysis: Glucose lowering effect (reduction of HbA_{1c}) of metformin versus acarbose (use a random-effect model instead)
