# Supplementary material for: Comparison of Glucose Lowering Effect of Metformin and Acarbose in Type 2 Diabetes Mellitus: A Meta-Analysis
Source: PLoS One. 2015 May 11;10(5):e0126704. doi: 10.1371/journal.pone.0126704 (PMC4427275; doi:10.1371/journal.pone.0126704)
Supplement: S2 Fig — (PDF) [file pone.0126704.s003.pdf]

Study or Subgroup	metformin Mean	SD	Total	placebo Mean	SD	Total	Weight	Mean Difference IV, Random, 95% CI
**1.1.1 Western**								
Chiasson 2001	-0.85	1.08	81	0.38	1.09	82	5.2%	-1.23 [-1.56, -0.90]
Damsbo 1998	-2.8	1.66	9	-3.3	3.57	9	1.1%	0.50 [-2.07, 3.07]
DeFronzo 1995	-1.3	1.31	143	0.4	2.65	146	4.9%	-1.70 [-2.18, -1.22]
Del Prato 2003	-1.02	1.63	250	0.48	1.86	127	5.1%	-1.50 [-1.88, -1.12]
Dornan 1991	-1.4	2.4	30	1.5	2.4	30	3.0%	-2.90 [-4.11, -1.69]
Goldstein 2007	-0.86	1.33	178	0.2	1.41	165	5.3%	-1.06 [-1.35, -0.77]
Goldstein 2007	-1.1	1.23	177	0.2	1.41	165	5.3%	-1.30 [-1.58, -1.02]
Haak 2012	-1.1	1.17	138	0.1	0.81	65	5.3%	-1.20 [-1.48, -0.92]
Haak 2012	-0.6	1.19	141	0.1	0.81	65	5.3%	-0.70 [-0.98, -0.42]
Horton 2004	-0.8	0.99	98	0.3	0.99	98	5.3%	-1.10 [-1.38, -0.82]
Hällsten 2002	-0.7	0.79	13	-0.2	0.41	14	4.9%	-0.50 [-0.98, -0.02]
Hällsten 2004	-0.6	0.99	9	-0.1	0.36	11	4.4%	-0.50 [-1.18, 0.18]
Karlsson 2005	-0.7	0.99	9	-0.1	0.36	11	4.4%	-0.60 [-1.28, 0.08]
Lee 1998	-1	1.4	24	-0.1	1.07	24	4.3%	-0.90 [-1.60, -0.20]
List 2009	-0.73	0.1	51	-0.18	0.1	44	5.6%	-0.55 [-0.59, -0.51]
Manzella 2004	-0.8	0.18	60	-0.2	0.29	60	5.6%	-0.60 [-0.69, -0.51]
Mather 2001	-0.3	1.16	28	-0.3	1.82	15	3.5%	0.00 [-1.02, 1.02]
Pirags 2012	-0.15	0.88	33	0.31	1.25	33	4.8%	-0.46 [-0.98, 0.06]
Uehara 2001	-0.7	1.41	11	-0.8	3.09	11	1.6%	0.10 [-1.91, 2.11]
Viljanen 2005	-0.6	0.89	12	-0.1	0.72	11	4.4%	-0.50 [-1.16, 0.16]
**Subtotal (95% CI)**			**1495**			**1186**	**89.4%**	**-0.92 [-1.09, -0.75]**
Heterogeneity: Tau² = 0.08; Chi² = 142.06, df = 19 (P < 0.00001); I² = 87%								
Test for overall effect: Z = 10.73 (P < 0.00001)								
**1.1.2 Eastern**								
Chakraborty 2011	-1.8	1.3	110	0.1	1.64	98	5.1%	-1.90 [-2.31, -1.49]
Wu 2001	-2.19	0.34	60	0.46	0.52	60	5.5%	-2.65 [-2.81, -2.49]
**Subtotal (95% CI)**			**170**			**158**	**10.6%**	**-2.30 [-3.03, -1.57]**
Heterogeneity: Tau² = 0.26; Chi² = 11.42, df = 1 (P = 0.0007); I² = 91%								
Test for overall effect: Z = 6.14 (P < 0.00001)								
**Total (95% CI)**			**1665**			**1344**	**100.0%**	**-1.05 [-1.36, -0.74]**
Heterogeneity: Tau² = 0.44; Chi² = 787.36, df = 21 (P < 0.00001); I² = 97%								
Test for overall effect: Z = 6.71 (P < 0.00001)								
Test for subgroup differences: Chi² = 12.86, df = 1 (P = 0.0003), I² = 92.2%								

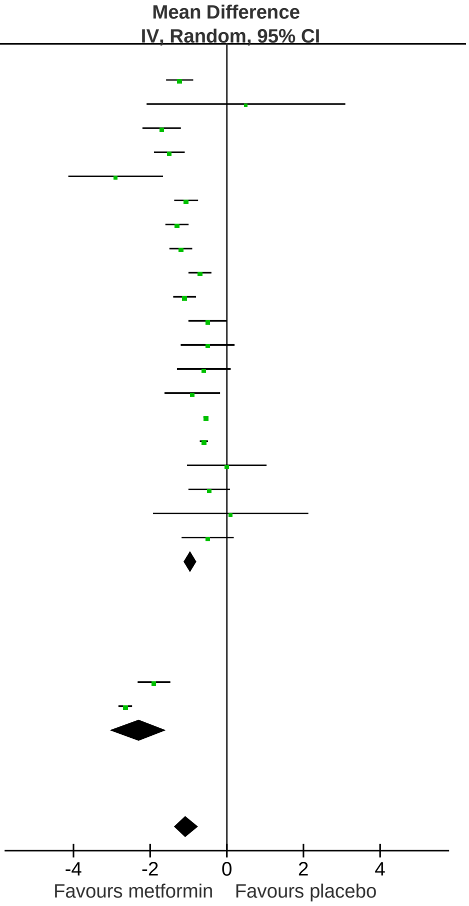

S2 Fig. Glucose lowering effect (reduction of HbA_{1c}) of metformin versus placebo (Western and Eastern as subgroups)
