# Supplementary material for: Comparison of Glucose Lowering Effect of Metformin and Acarbose in Type 2 Diabetes Mellitus: A Meta-Analysis
Source: PLoS One. 2015 May 11;10(5):e0126704. doi: 10.1371/journal.pone.0126704 (PMC4427275; doi:10.1371/journal.pone.0126704)
Supplement: S3 Fig — (PDF) [file pone.0126704.s004.pdf]

| Study or Subgroup | metformin Mean | metformin SD | metformin Total | placebo Mean | placebo SD | placebo Total | Weight | Mean Difference IV, Fixed, 95% CI |
|---|---|---|---|---|---|---|---|---|
| **1.1.1 Metformin 1000 mg/d** | | | | | | | | |
| Goldstein 2007 | -0.86 | 1.33 | 178 | 0.2 | 1.41 | 165 | 1.3% | -1.06 [-1.35, -0.77] |
| Haak 2012 | -0.6 | 1.19 | 141 | 0.1 | 0.81 | 65 | 1.4% | -0.70 [-0.98, -0.42] |
| Mather 2001 | -0.3 | 1.16 | 28 | -0.3 | 1.82 | 15 | 0.1% | 0.00 [-1.02, 1.02] |
| Uehara 2001 | -0.7 | 1.41 | 11 | -0.8 | 3.09 | 11 | 0.0% | 0.10 [-1.91, 2.11] |
| Wu 2001 | -2.19 | 0.34 | 60 | 0.46 | 0.52 | 60 | 4.5% | -2.65 [-2.81, -2.49] |
| **Subtotal (95% CI)** | | | **418** | | | **316** | **7.4%** | **-1.94 [-2.06, -1.82]** |
| Heterogeneity: Chi² = 207.90, df = 4 (P < 0.00001); I² = 98% | | | | | | | | |
| Test for overall effect: Z = 30.97 (P < 0.00001) | | | | | | | | |
| **1.1.2 Metformin 1500 mg/d** | | | | | | | | |
| Chiasson 2001 | -0.85 | 1.08 | 81 | 0.38 | 1.09 | 82 | 1.0% | -1.23 [-1.56, -0.90] |
| Horton 2004 | -0.8 | 0.99 | 98 | 0.3 | 0.99 | 98 | 1.4% | -1.10 [-1.38, -0.82] |
| List 2009 | -0.73 | 0.1 | 51 | -0.18 | 0.1 | 44 | 68.5% | -0.55 [-0.59, -0.51] |
| **Subtotal (95% CI)** | | | **230** | | | **224** | **70.9%** | **-0.57 [-0.61, -0.53]** |
| Heterogeneity: Chi² = 30.06, df = 2 (P < 0.00001); I² = 93% | | | | | | | | |
| Test for overall effect: Z = 28.24 (P < 0.00001) | | | | | | | | |
| **1.1.3 Metformin 1700mg/d** | | | | | | | | |
| Chakraborty 2011 | -1.8 | 1.3 | 110 | 0.1 | 1.64 | 98 | 0.7% | -1.90 [-2.31, -1.49] |
| Lee 1998 | -1 | 1.4 | 24 | -0.1 | 1.07 | 24 | 0.2% | -0.90 [-1.60, -0.20] |
| Manzella 2004 | -0.8 | 0.18 | 60 | -0.2 | 0.29 | 60 | 14.9% | -0.60 [-0.69, -0.51] |
| Pirags 2012 | -0.15 | 0.88 | 33 | 0.31 | 1.25 | 33 | 0.4% | -0.46 [-0.98, 0.06] |
| **Subtotal (95% CI)** | | | **227** | | | **215** | **16.2%** | **-0.65 [-0.74, -0.57]** |
| Heterogeneity: Chi² = 38.77, df = 3 (P < 0.00001); I² = 92% | | | | | | | | |
| Test for overall effect: Z = 15.50 (P < 0.00001) | | | | | | | | |
| **1.1.4 Metformin 2000mg/d** | | | | | | | | |
| Goldstein 2007 | -1.1 | 1.23 | 177 | 0.2 | 1.41 | 165 | 1.4% | -1.30 [-1.58, -1.02] |
| Haak 2012 | -1.1 | 1.17 | 138 | 0.1 | 0.81 | 65 | 1.4% | -1.20 [-1.48, -0.92] |
| Hällsten 2002 | -0.7 | 0.79 | 13 | -0.2 | 0.41 | 14 | 0.5% | -0.50 [-0.98, -0.02] |
| Hällsten 2004 | -0.6 | 0.99 | 9 | -0.1 | 0.36 | 11 | 0.2% | -0.50 [-1.18, 0.18] |
| Karlsson 2005 | -0.7 | 0.99 | 9 | -0.1 | 0.36 | 11 | 0.2% | -0.60 [-1.28, 0.08] |
| Viljanen 2005 | -0.6 | 0.89 | 12 | -0.1 | 0.72 | 11 | 0.3% | -0.50 [-1.16, 0.16] |
| **Subtotal (95% CI)** | | | **358** | | | **277** | **4.1%** | **-1.03 [-1.20, -0.87]** |
| Heterogeneity: Chi² = 16.01, df = 5 (P = 0.007); I² = 69% | | | | | | | | |
| Test for overall effect: Z = 12.22 (P < 0.00001) | | | | | | | | |
| **1.1.5 Metformin 2550mg/d** | | | | | | | | |
| DeFronzo 1995 | -1.3 | 1.31 | 143 | 0.4 | 2.65 | 146 | 0.5% | -1.70 [-2.18, -1.22] |
| Del Prato 2003 | -1.02 | 1.63 | 250 | 0.48 | 1.86 | 127 | 0.8% | -1.50 [-1.88, -1.12] |
| **Subtotal (95% CI)** | | | **393** | | | **273** | **1.2%** | **-1.58 [-1.88, -1.28]** |
| Heterogeneity: Chi² = 0.41, df = 1 (P = 0.52); I² = 0% | | | | | | | | |
| Test for overall effect: Z = 10.35 (P < 0.00001) | | | | | | | | |
| **1.1.6 Metformin 3000mg/d** | | | | | | | | |
| Damsbo 1998 | -2.8 | 1.66 | 9 | -3.3 | 3.57 | 9 | 0.0% | 0.50 [-2.07, 3.07] |
| Dornan 1991 | -1.4 | 2.4 | 30 | 1.5 | 2.4 | 30 | 0.1% | -2.90 [-4.11, -1.69] |
| **Subtotal (95% CI)** | | | **39** | | | **39** | **0.1%** | **-2.28 [-3.38, -1.18]** |
| Heterogeneity: Chi² = 5.49, df = 1 (P = 0.02); I² = 82% | | | | | | | | |
| Test for overall effect: Z = 4.07 (P < 0.0001) | | | | | | | | |
| **Total (95% CI)** | | | **1665** | | | **1344** | **100.0%** | **-0.72 [-0.75, -0.69]** |
| Heterogeneity: Chi² = 787.36, df = 21 (P < 0.00001); I² = 97% | | | | | | | | |
| Test for overall effect: Z = 42.20 (P < 0.00001) | | | | | | | | |
| Test for subgroup differences: Chi² = 488.73, df = 5 (P < 0.00001), I² = 99.0% | | | | | | | | |

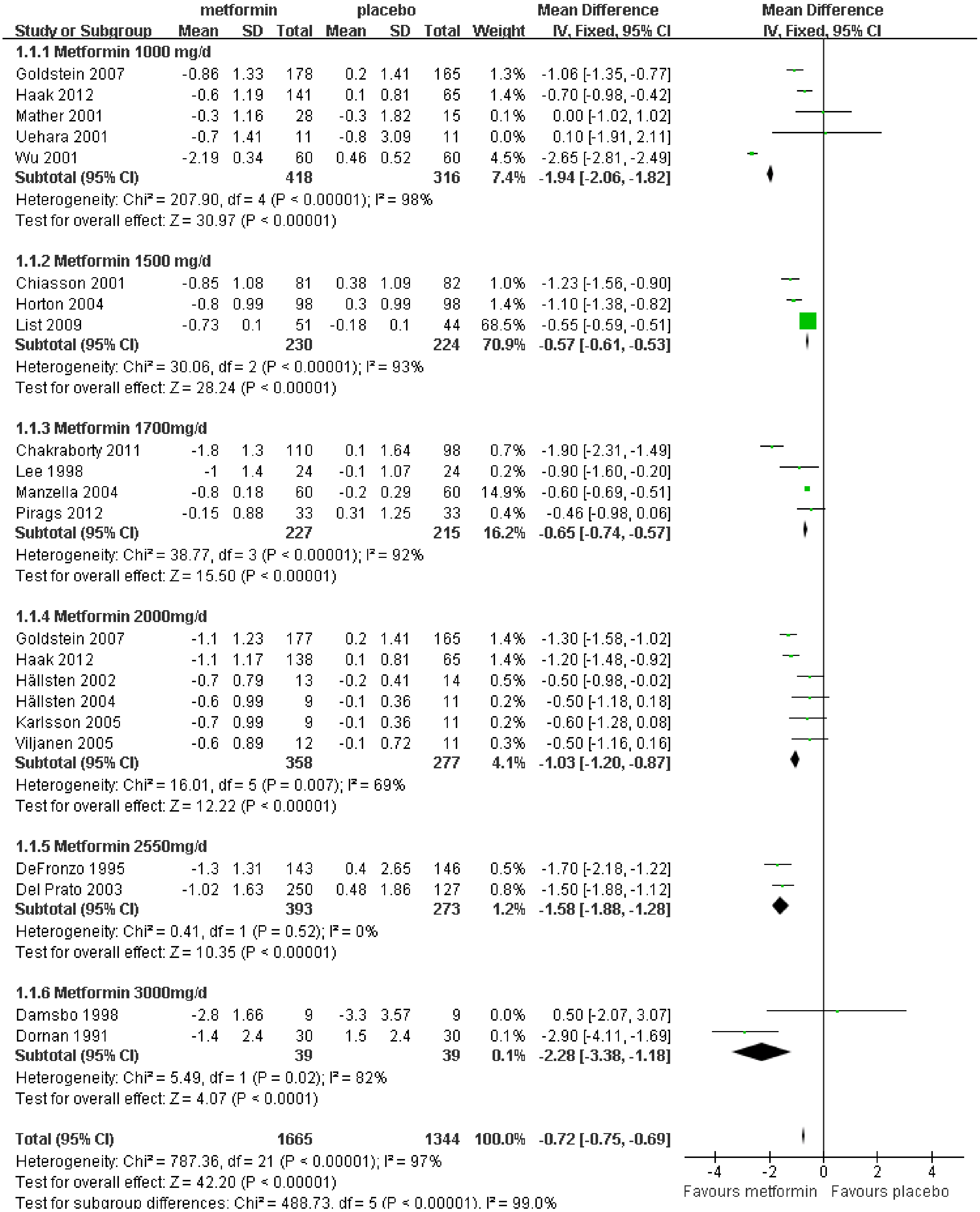


**S3 Fig. Sensitivity analysis: Glucose lowering effect (reduction of $HbA_{1c}$) of metformin versus placebo (use a fixed-effect model instead)**
