# Supplementary material for: Comparison of Glucose Lowering Effect of Metformin and Acarbose in Type 2 Diabetes Mellitus: A Meta-Analysis
Source: PLoS One. 2015 May 11;10(5):e0126704. doi: 10.1371/journal.pone.0126704 (PMC4427275; doi:10.1371/journal.pone.0126704)
Supplement: S4 Fig — (PDF) [file pone.0126704.s005.pdf]

	metformin			placebo				Mean Difference
Study or Subgroup	Mean	SD	Total	Mean	SD	Total	Weight	IV, Random, 95% CI
**1.1.1 Metformin 1000 mg/d**								
Goldstein 2007	-0.86	1.33	178	0.2	1.41	165	5.6%	-1.06 [-1.35, -0.77]
Haak 2012	-0.6	1.19	141	0.1	0.81	65	5.6%	-0.70 [-0.98, -0.42]
Mather 2001	-0.3	1.16	28	-0.3	1.82	15	3.7%	0.00 [-1.02, 1.02]
Uehara 2001	-0.7	1.41	11	-0.8	3.09	11	1.7%	0.10 [-1.91, 2.11]
Wu 2001	-2.19	0.34	60	0.46	0.52	60	5.8%	-2.65 [-2.81, -2.49]
**Subtotal (95% CI)**			**418**			**316**	**22.3%**	**-0.98 [-2.10, 0.13]**
Heterogeneity: Tau² = 1.43; Chi² = 207.90, df = 4 (P < 0.00001); I² = 98%								
Test for overall effect: Z = 1.72 (P = 0.08)								
**1.1.2 Metformin 1500 mg/d**								
Chiasson 2001	-0.85	1.08	81	0.38	1.09	82	5.5%	-1.23 [-1.56, -0.90]
Horton 2004	-0.8	0.99	98	0.3	0.99	98	5.6%	-1.10 [-1.38, -0.82]
List 2009	-0.73	0.1	51	-0.18	0.1	44	5.8%	-0.55 [-0.59, -0.51]
**Subtotal (95% CI)**			**230**			**224**	**16.9%**	**-0.94 [-1.43, -0.45]**
Heterogeneity: Tau² = 0.17; Chi² = 30.06, df = 2 (P < 0.00001); I² = 93%								
Test for overall effect: Z = 3.78 (P = 0.0002)								
**1.1.3 Metformin 1700mg/d**								
Chakraborty 2011	-1.8	1.3	110	0.1	1.64	98	5.3%	-1.90 [-2.31, -1.49]
Lee 1998	-1	1.4	24	-0.1	1.07	24	4.5%	-0.90 [-1.60, -0.20]
Manzella 2004	-0.8	0.18	60	-0.2	0.29	60	5.8%	-0.60 [-0.69, -0.51]
Pirags 2012	-0.15	0.88	33	0.31	1.25	33	5.0%	-0.46 [-0.98, 0.06]
**Subtotal (95% CI)**			**227**			**215**	**20.8%**	**-0.97 [-1.64, -0.29]**
Heterogeneity: Tau² = 0.42; Chi² = 38.77, df = 3 (P < 0.00001); I² = 92%								
Test for overall effect: Z = 2.80 (P = 0.005)								
**1.1.4 Metformin 2000mg/d**								
Goldstein 2007	-1.1	1.23	177	0.2	1.41	165	5.6%	-1.30 [-1.58, -1.02]
Haak 2012	-1.1	1.17	138	0.1	0.81	65	5.6%	-1.20 [-1.48, -0.92]
Hällsten 2004	-0.6	0.99	9	-0.1	0.36	11	4.6%	-0.50 [-1.18, 0.18]
Karlsson 2005	-0.7	0.99	9	-0.1	0.36	11	4.6%	-0.60 [-1.28, 0.08]
Viljanen 2005	-0.6	0.89	12	-0.1	0.72	11	4.7%	-0.50 [-1.16, 0.16]
**Subtotal (95% CI)**			**345**			**263**	**25.1%**	**-0.94 [-1.27, -0.61]**
Heterogeneity: Tau² = 0.08; Chi² = 10.68, df = 4 (P = 0.03); I² = 63%								
Test for overall effect: Z = 5.56 (P < 0.00001)								
**1.1.5 Metformin 2550mg/d**								
DeFronzo 1995	-1.3	1.31	143	0.4	2.65	146	5.2%	-1.70 [-2.18, -1.22]
Del Prato 2003	-1.02	1.63	250	0.48	1.86	127	5.4%	-1.50 [-1.88, -1.12]
**Subtotal (95% CI)**			**393**			**273**	**10.5%**	**-1.58 [-1.88, -1.28]**
Heterogeneity: Tau² = 0.00; Chi² = 0.41, df = 1 (P = 0.52); I² = 0%								
Test for overall effect: Z = 10.35 (P < 0.00001)								
**1.1.6 Metformin 3000mg/d**								
Damsbo 1998	-2.8	1.66	9	-3.3	3.57	9	1.2%	0.50 [-2.07, 3.07]
Dornan 1991	-1.4	2.4	30	1.5	2.4	30	3.1%	-2.90 [-4.11, -1.69]
**Subtotal (95% CI)**			**39**			**39**	**4.3%**	**-1.40 [-4.71, 1.91]**
Heterogeneity: Tau² = 4.73; Chi² = 5.49, df = 1 (P = 0.02); I² = 82%								
Test for overall effect: Z = 0.83 (P = 0.41)								
**Total (95% CI)**			**1652**			**1330**	**100.0%**	**-1.08 [-1.40, -0.76]**
Heterogeneity: Tau² = 0.45; Chi² = 786.56, df = 20 (P < 0.00001); I² = 97%								
Test for overall effect: Z = 6.68 (P < 0.00001)								
Test for subgroup differences: Chi² = 10.19, df = 5 (P = 0.07), I² = 50.9%								

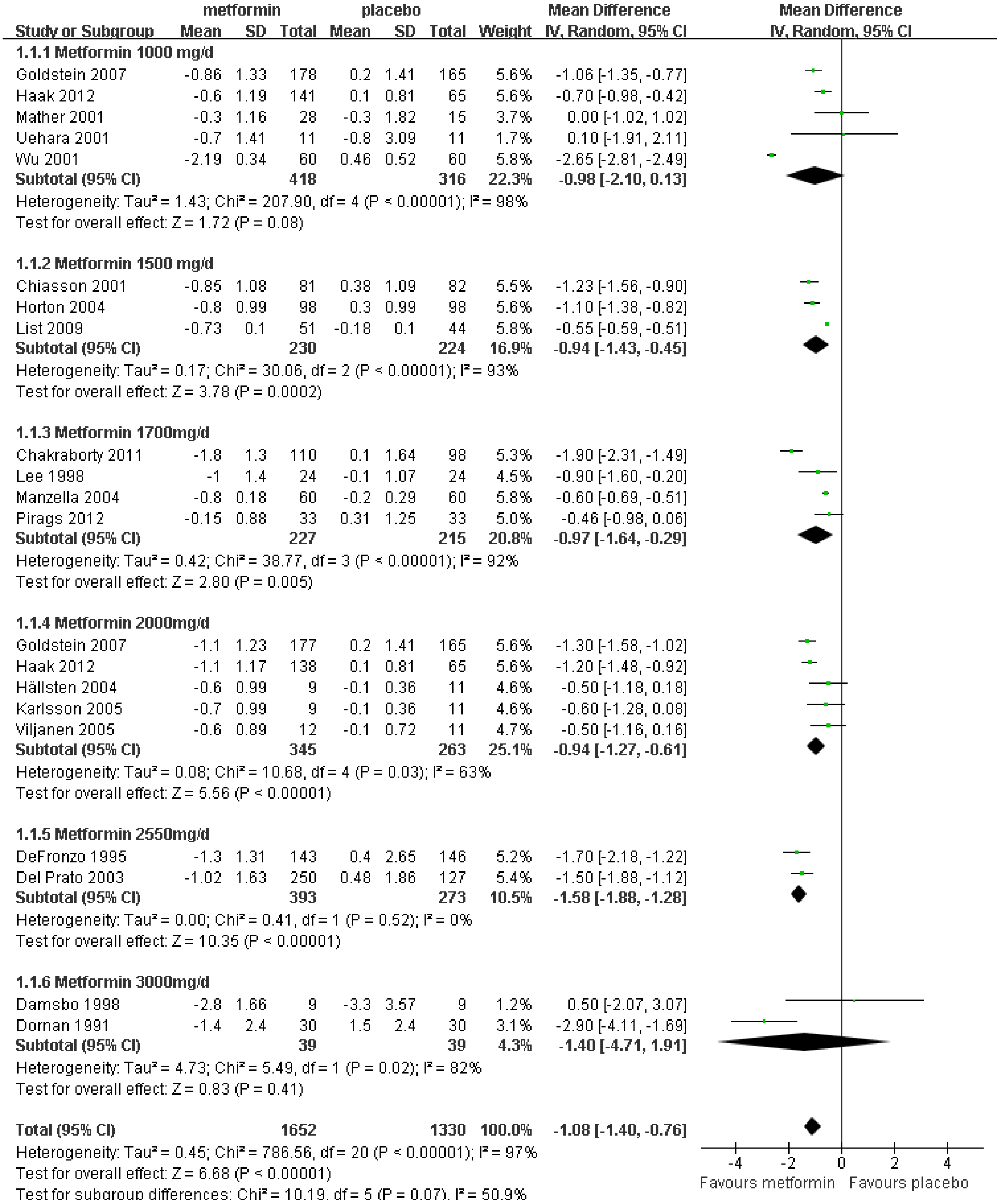

S4 Fig. Sensitivity analysis: Glucose lowering effect (reduction of HbA_{1c}) of metformin versus placebo (remove one low-quality study [34] from the original data set)
