# Supplementary material for: Comparison of Glucose Lowering Effect of Metformin and Acarbose in Type 2 Diabetes Mellitus: A Meta-Analysis
Source: PLoS One. 2015 May 11;10(5):e0126704. doi: 10.1371/journal.pone.0126704 (PMC4427275; doi:10.1371/journal.pone.0126704)
Supplement: S5 Fig — (PDF) [file pone.0126704.s006.pdf]

| | acarbose | | | placebo | | | | Mean Difference | Mean Difference |
|---|---|---|---|---|---|---|---|---|---|
| **Study or Subgroup** | **Mean** | **SD** | **Total** | **Mean** | **SD** | **Total** | **Weight** | **IV, Random, 95% CI** | **IV, Random, 95% CI** |
| **2.1.1 Western** | | | | | | | | | |
| Buchanan 1988 | 1.1 | 3.53 | 9 | 1.6 | 3.86 | 11 | 0.3% | -0.50 [-3.74, 2.74] | |
| Derosa 2011 | -1.1 | 0.57 | 88 | -0.3 | 0.47 | 87 | 10.1% | -0.80 [-0.95, -0.65] | |
| Fischer 1998 | -0.05 | 1.24 | 86 | 0.37 | 1.26 | 81 | 7.2% | -0.42 [-0.80, -0.04] | |
| Fischer 1998 | -0.72 | 1.26 | 87 | 0.37 | 1.26 | 81 | 7.1% | -1.09 [-1.47, -0.71] | |
| Fischer 1998 | -0.45 | 1.26 | 78 | 0.37 | 1.26 | 81 | 7.0% | -0.82 [-1.21, -0.43] | |
| Fischer 1998 | -0.44 | 1.25 | 88 | 0.37 | 1.26 | 81 | 7.2% | -0.81 [-1.19, -0.43] | |
| Fischer 2003 | 0 | 1.63 | 25 | 0.7 | 1.43 | 25 | 3.0% | -0.70 [-1.55, 0.15] | |
| Hanefeld 1991 | -0.65 | 1.37 | 47 | -0.08 | 1.36 | 47 | 5.2% | -0.57 [-1.12, -0.02] | |
| Hanefeld 2002 | 0.4 | 2.13 | 11 | 0.9 | 1.86 | 8 | 0.8% | -0.50 [-2.30, 1.30] | |
| Josse 2003 | -0.3 | 0.96 | 93 | 0.3 | 0.99 | 99 | 8.6% | -0.60 [-0.88, -0.32] | |
| Kirkman 2006 | -0.19 | 0.76 | 81 | -0.1 | 0.67 | 76 | 9.3% | -0.09 [-0.31, 0.13] | |
| Meneilly 2000 | -0.4 | 0.47 | 22 | 0.4 | 0.96 | 23 | 6.4% | -0.80 [-1.24, -0.36] | |
| Rosenbaum 2002 | -0.8 | 1.98 | 20 | 0 | 2.25 | 20 | 1.5% | -0.80 [-2.11, 0.51] | |
| Scott 1999 | -0.14 | 0.91 | 41 | 0.25 | 1.15 | 42 | 6.3% | -0.39 [-0.84, 0.06] | |
| Wolever 1997 | -0.86 | 1.45 | 25 | 0.02 | 0.97 | 29 | 4.1% | -0.88 [-1.55, -0.21] | |
| **Subtotal (95% CI)** | | | **801** | | | **791** | **84.1%** | **-0.65 [-0.83, -0.46]** | |
| Heterogeneity: Tau² = 0.06; Chi² = 37.97, df = 14 (P = 0.0005); I² = 63% | | | | | | | | | |
| Test for overall effect: Z = 6.98 (P < 0.00001) | | | | | | | | | |
| **2.1.3 Eastern** | | | | | | | | | |
| Chan 1998 | -0.7 | 1.22 | 59 | -0.27 | 1.09 | 62 | 6.7% | -0.43 [-0.84, -0.02] | |
| Hotta 1993 | -1.4 | 2.65 | 19 | -0.4 | 2.12 | 18 | 1.1% | -1.00 [-2.54, 0.54] | |
| Hwu 2003 | -0.5 | 1.3 | 54 | 0.2 | 1.2 | 53 | 6.0% | -0.70 [-1.17, -0.23] | |
| Wu 2003 | -3.2 | 3 | 80 | -0.9 | 4 | 80 | 2.0% | -2.30 [-3.40, -1.20] | |
| **Subtotal (95% CI)** | | | **212** | | | **213** | **15.9%** | **-0.94 [-1.59, -0.30]** | |
| Heterogeneity: Tau² = 0.27; Chi² = 9.97, df = 3 (P = 0.02); I² = 70% | | | | | | | | | |
| Test for overall effect: Z = 2.86 (P = 0.004) | | | | | | | | | |
| **Total (95% CI)** | | | **1013** | | | **1004** | **100.0%** | **-0.67 [-0.85, -0.50]** | |
| Heterogeneity: Tau² = 0.07; Chi² = 48.08, df = 18 (P = 0.0001); I² = 63% | | | | | | | | | |
| Test for overall effect: Z = 7.67 (P < 0.00001) | | | | | | | | | |
| Test for subgroup differences: Chi² = 0.75, df = 1 (P = 0.39), I² = 0% | | | | | | | | | |

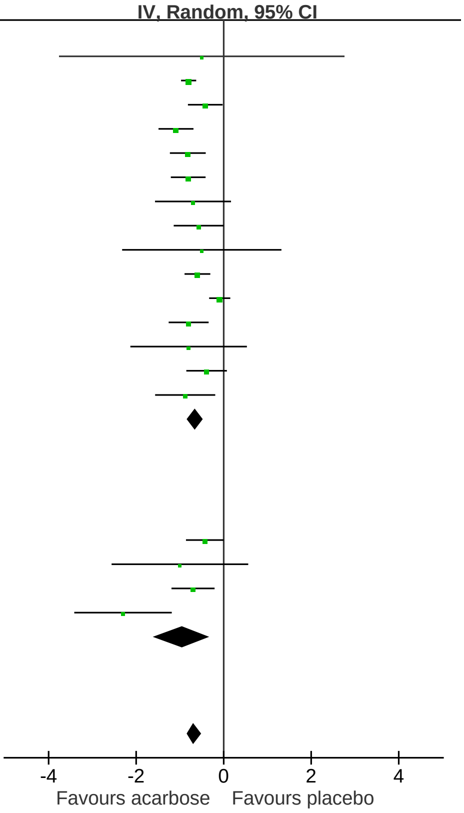


**S5 Fig. Glucose lowering effect (reduction of $HbA_{1c}$) of acarbose versus placebo (Western and Eastern as subgroups)**
