# Supplementary material for: Comparison of Glucose Lowering Effect of Metformin and Acarbose in Type 2 Diabetes Mellitus: A Meta-Analysis
Source: PLoS One. 2015 May 11;10(5):e0126704. doi: 10.1371/journal.pone.0126704 (PMC4427275; doi:10.1371/journal.pone.0126704)
Supplement: S7 Fig — (PDF) [file pone.0126704.s008.pdf]

Study or Subgroup	acarbose Mean	acarbose SD	acarbose Total	placebo Mean	placebo SD	placebo Total	Weight	Mean Difference IV, Random, 95% CI
**2.1.1 Acarbose 75mg/d**								
Fischer 1998	-0.05	1.24	86	0.37	1.26	81	8.1%	-0.42 [-0.80, -0.04]
**Subtotal (95% CI)**			**86**			**81**	**8.1%**	**-0.42 [-0.80, -0.04]**
Heterogeneity: Not applicable								
Test for overall effect: Z = 2.17 (P = 0.03)								
**2.1.3 Acarbose 150mg/d**								
Fischer 1998	-0.44	1.25	88	0.37	1.26	81	8.1%	-0.81 [-1.19, -0.43]
**Subtotal (95% CI)**			**88**			**81**	**8.1%**	**-0.81 [-1.19, -0.43]**
Heterogeneity: Not applicable								
Test for overall effect: Z = 4.19 (P < 0.0001)								
**2.1.4 Acarbose 300mg/d**								
Chan 1998	-0.7	1.22	59	-0.27	1.09	62	7.7%	-0.43 [-0.84, -0.02]
Derosa 2011	-1.1	0.57	88	-0.3	0.47	87	10.9%	-0.80 [-0.95, -0.65]
Fischer 1998	-0.45	1.26	78	0.37	1.26	81	8.0%	-0.82 [-1.21, -0.43]
Hanefeld 1991	-0.65	1.37	47	-0.08	1.36	47	6.0%	-0.57 [-1.12, -0.02]
Hotta 1993	-1.4	2.65	19	-0.4	2.12	18	1.4%	-1.00 [-2.54, 0.54]
Hwu 2003	-0.5	1.3	54	0.2	1.2	53	6.9%	-0.70 [-1.17, -0.23]
Josse 2003	-0.3	0.96	93	0.3	0.99	99	9.5%	-0.60 [-0.88, -0.32]
Kirkman 2006	-0.19	0.76	81	-0.1	0.67	76	10.2%	-0.09 [-0.31, 0.13]
Scott 1999	-0.14	0.91	41	0.25	1.15	42	7.3%	-0.39 [-0.84, 0.06]
Wolever 1997	-0.86	1.45	25	0.02	0.97	29	4.9%	-0.88 [-1.55, -0.21]
Wu 2003	-3.2	3	80	-0.9	4	80	2.5%	-2.30 [-3.40, -1.20]
**Subtotal (95% CI)**			**665**			**674**	**75.3%**	**-0.64 [-0.87, -0.40]**
Heterogeneity: Tau² = 0.10; Chi² = 39.45, df = 10 (P < 0.0001); I² = 75%								
Test for overall effect: Z = 5.34 (P < 0.00001)								
**2.1.5 Acarbose 400mg/d**								
Buchanan 1988	1.1	3.53	9	1.6	3.86	11	0.3%	-0.50 [-3.74, 2.74]
**Subtotal (95% CI)**			**9**			**11**	**0.3%**	**-0.50 [-3.74, 2.74]**
Heterogeneity: Not applicable								
Test for overall effect: Z = 0.30 (P = 0.76)								
**2.1.6 Acarbose 600mg/d**								
Fischer 1998	-0.72	1.26	87	0.37	1.26	81	8.1%	-1.09 [-1.47, -0.71]
**Subtotal (95% CI)**			**87**			**81**	**8.1%**	**-1.09 [-1.47, -0.71]**
Heterogeneity: Not applicable								
Test for overall effect: Z = 5.60 (P < 0.00001)								
**Total (95% CI)**			**935**			**928**	**100.0%**	**-0.67 [-0.86, -0.47]**
Heterogeneity: Tau² = 0.08; Chi² = 47.42, df = 14 (P < 0.0001); I² = 70%								
Test for overall effect: Z = 6.72 (P < 0.00001)								
Test for subgroup differences: Chi² = 6.68, df = 4 (P = 0.15), I² = 40.2%								

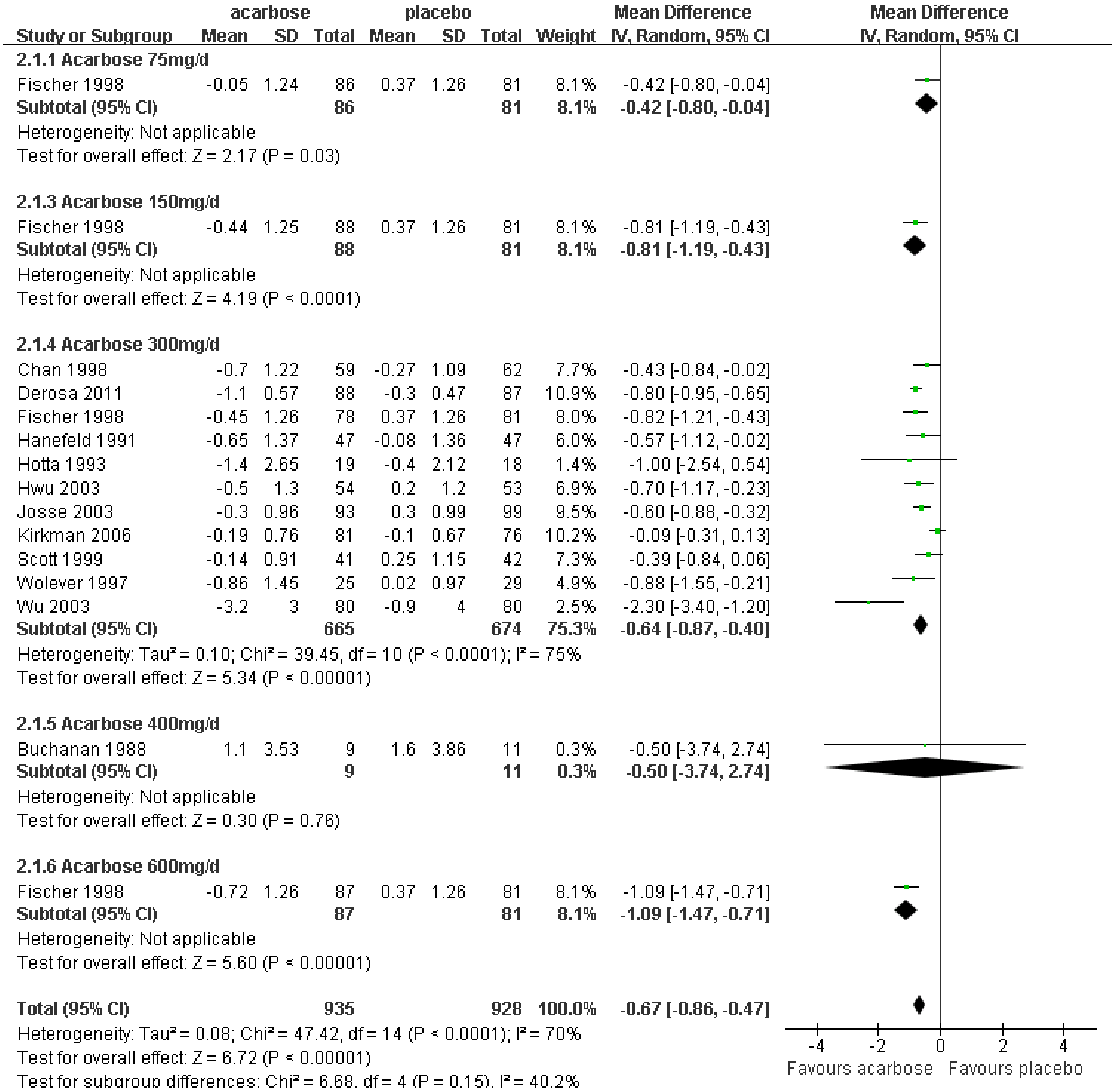

S7 Fig. Sensitivity analysis: Glucose lowering effect (reduction of HbA_{1c}) of acarbose versus placebo (remove four low-quality studies [45, 47, 52, 53] from the original data set)
