# Supplementary material for: Comparison of Glucose Lowering Effect of Metformin and Acarbose in Type 2 Diabetes Mellitus: A Meta-Analysis
Source: PLoS One. 2015 May 11;10(5):e0126704. doi: 10.1371/journal.pone.0126704 (PMC4427275; doi:10.1371/journal.pone.0126704)
Supplement: S8 Fig — (PDF) [file pone.0126704.s009.pdf]

Study or Subgroup	metformin Mean	metformin SD	metformin Total	sulphonylurea Mean	sulphonylurea SD	sulphonylurea Total	Weight	Mean Difference IV, Random, 95% CI
**3.1.1 Western**								
Amador-Licona 2000	-0.9	1.39	28	-0.8	1.31	23	3.7%	-0.10 [-0.84, 0.64]
Charpentier 2001	0.07	1.45	75	0.27	1.42	150	5.3%	-0.20 [-0.60, 0.20]
DeFronzo 1995	-0.4	1.45	210	0.2	1.45	209	5.9%	-0.60 [-0.88, -0.32]
Formoso 2008	-2.2	0.04	13	-1.4	0.03	13	6.5%	-0.80 [-0.83, -0.77]
Goldstein 2003	-0.2	1.16	71	-0.4	1.1	79	5.5%	0.20 [-0.16, 0.56]
Lawrence 2004	-1.14	0.84	20	-1.21	0.84	20	4.7%	0.07 [-0.45, 0.59]
Tessier 1999	-1	1.56	18	-1	1.87	18	2.4%	0.00 [-1.13, 1.13]
**Subtotal (95% CI)**			**435**			**512**	**33.8%**	**-0.26 [-0.63, 0.12]**
Heterogeneity: Tau² = 0.20; Chi² = 54.93, df = 6 (P < 0.00001); I² = 89%								
Test for overall effect: Z = 1.33 (P = 0.18)								
**3.1.2 Eastern**								
Chen 2004	-1.69	1.94	32	-1.7	1.89	30	2.9%	0.01 [-0.94, 0.96]
Chen 2012	-1.3	0.8	26	-0.9	0.8	27	5.2%	-0.40 [-0.83, 0.03]
Chien 2007	0.09	0.37	17	0.52	0.24	17	6.1%	-0.43 [-0.64, -0.22]
Jia 2005	-1.7	2.02	29	-1.1	1.56	31	3.0%	-0.60 [-1.52, 0.32]
Ling 2003	-2.96	0.94	44	-2.95	1.11	50	5.2%	-0.01 [-0.42, 0.40]
Liu 2013	-1.59	1.45	38	-1.28	0.55	35	4.8%	-0.31 [-0.81, 0.19]
Ning 2006	-2.02	2.13	50	-1.5	1.98	51	3.4%	-0.52 [-1.32, 0.28]
Ramachandran 2004	-1.4	2.69	21	-2.5	2.18	18	1.5%	1.10 [-0.43, 2.63]
Tang 2004	-0.5	1.48	29	-0.7	1.51	33	3.7%	0.20 [-0.55, 0.95]
Wang 2011	-1.7	1.5	68	-0.9	1.61	68	4.7%	-0.80 [-1.32, -0.28]
Xu 2006	-2.96	0.94	44	-3	1.11	50	5.2%	0.04 [-0.37, 0.45]
Xuan 2012	-2.31	2.23	35	-3	3.02	35	2.1%	0.69 [-0.55, 1.93]
Yamanouchi 2005	-2.1	0.96	39	-2.1	0.89	37	5.2%	0.00 [-0.42, 0.42]
Yao 2008	-0.09	1.3	58	-0.5	1.28	59	5.0%	0.41 [-0.06, 0.88]
Yao 2010	-1.5	1.72	118	-1.3	1.7	118	5.1%	-0.20 [-0.64, 0.24]
Zhang 2009	-0.31	1.19	10	-0.2	0.79	10	3.1%	-0.11 [-1.00, 0.78]
**Subtotal (95% CI)**			**658**			**669**	**66.2%**	**-0.16 [-0.34, 0.02]**
Heterogeneity: Tau² = 0.05; Chi² = 27.55, df = 15 (P = 0.02); I² = 46%								
Test for overall effect: Z = 1.70 (P = 0.09)								
**Total (95% CI)**			**1093**			**1181**	**100.0%**	**-0.18 [-0.39, 0.04]**
Heterogeneity: Tau² = 0.19; Chi² = 166.38, df = 22 (P < 0.00001); I² = 87%								
Test for overall effect: Z = 1.59 (P = 0.11)								
Test for subgroup differences: Chi² = 0.21, df = 1 (P = 0.65), I² = 0%								

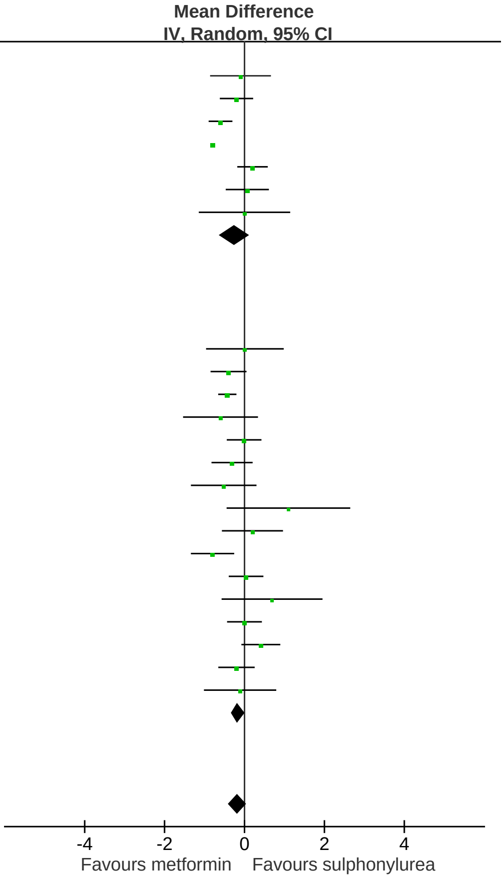

S8 Fig. Glucose lowering effect (reduction of HbA_{1c}) of metformin versus sulphonylurea (Western and Eastern as subgroups)
