# Supplementary material for: Comparison of Glucose Lowering Effect of Metformin and Acarbose in Type 2 Diabetes Mellitus: A Meta-Analysis
Source: PLoS One. 2015 May 11;10(5):e0126704. doi: 10.1371/journal.pone.0126704 (PMC4427275; doi:10.1371/journal.pone.0126704)
Supplement: S9 Fig — (PDF) [file pone.0126704.s010.pdf]

Study or Subgroup	metformin Mean	metformin SD	metformin Total	sulphonylurea Mean	sulphonylurea SD	sulphonylurea Total	Weight	Mean Difference IV, Random, 95% CI
**3.1.1 Metformin ≦1000mg/d**								
Chien 2007	0.09	0.37	17	0.52	0.24	17	14.4%	-0.43 [-0.64, -0.22]
Yamanouchi 2005	-2.1	0.96	39	-2.1	0.89	37	10.8%	0.00 [-0.42, 0.42]
**Subtotal (95% CI)**			**56**			**54**	**25.2%**	**-0.25 [-0.67, 0.16]**
Heterogeneity: Tau² = 0.06; Chi² = 3.27, df = 1 (P = 0.07); I² = 69%								
Test for overall effect: Z = 1.20 (P = 0.23)								
**3.1.2 Metformin 1500mg/d**								
Jia 2005	-1.7	2.02	29	-1.1	1.56	31	4.6%	-0.60 [-1.52, 0.32]
Wang 2011	-1.7	1.5	68	-0.9	1.61	68	9.0%	-0.80 [-1.32, -0.28]
Yao 2008	-0.09	1.3	58	-0.5	1.28	59	9.9%	0.41 [-0.06, 0.88]
Yao 2010	-1.5	1.72	118	-1.3	1.7	118	10.4%	-0.20 [-0.64, 0.24]
Zhang 2009	-0.31	1.19	10	-0.2	0.79	10	4.9%	-0.11 [-1.00, 0.78]
**Subtotal (95% CI)**			**283**			**286**	**38.7%**	**-0.23 [-0.70, 0.24]**
Heterogeneity: Tau² = 0.18; Chi² = 12.35, df = 4 (P = 0.01); I² = 68%								
Test for overall effect: Z = 0.97 (P = 0.33)								
**3.1.3 Metformin 2000mg/d**								
Goldstein 2003	-0.2	1.16	71	-0.4	1.1	79	11.7%	0.20 [-0.16, 0.56]
**Subtotal (95% CI)**			**71**			**79**	**11.7%**	**0.20 [-0.16, 0.56]**
Heterogeneity: Not applicable								
Test for overall effect: Z = 1.08 (P = 0.28)								
**3.1.4 Metformin 2550mg/d**								
Charpentier 2001	0.07	1.45	75	0.27	1.42	150	11.1%	-0.20 [-0.60, 0.20]
DeFronzo 1995	-0.4	1.45	210	0.2	1.45	209	13.3%	-0.60 [-0.88, -0.32]
**Subtotal (95% CI)**			**285**			**359**	**24.3%**	**-0.43 [-0.82, -0.04]**
Heterogeneity: Tau² = 0.05; Chi² = 2.60, df = 1 (P = 0.11); I² = 62%								
Test for overall effect: Z = 2.15 (P = 0.03)								
**3.1.5 Metformin 3000mg/d**								
**Subtotal (95% CI)**			**0**			**0**		**Not estimable**
Heterogeneity: Not applicable								
Test for overall effect: Not applicable								
**Total (95% CI)**			**695**			**778**	**100.0%**	**-0.23 [-0.46, 0.01]**
Heterogeneity: Tau² = 0.09; Chi² = 28.58, df = 9 (P = 0.0008); I² = 69%								
Test for overall effect: Z = 1.90 (P = 0.06)								
Test for subgroup differences: Chi² = 5.88, df = 3 (P = 0.12), I² = 49.0%								

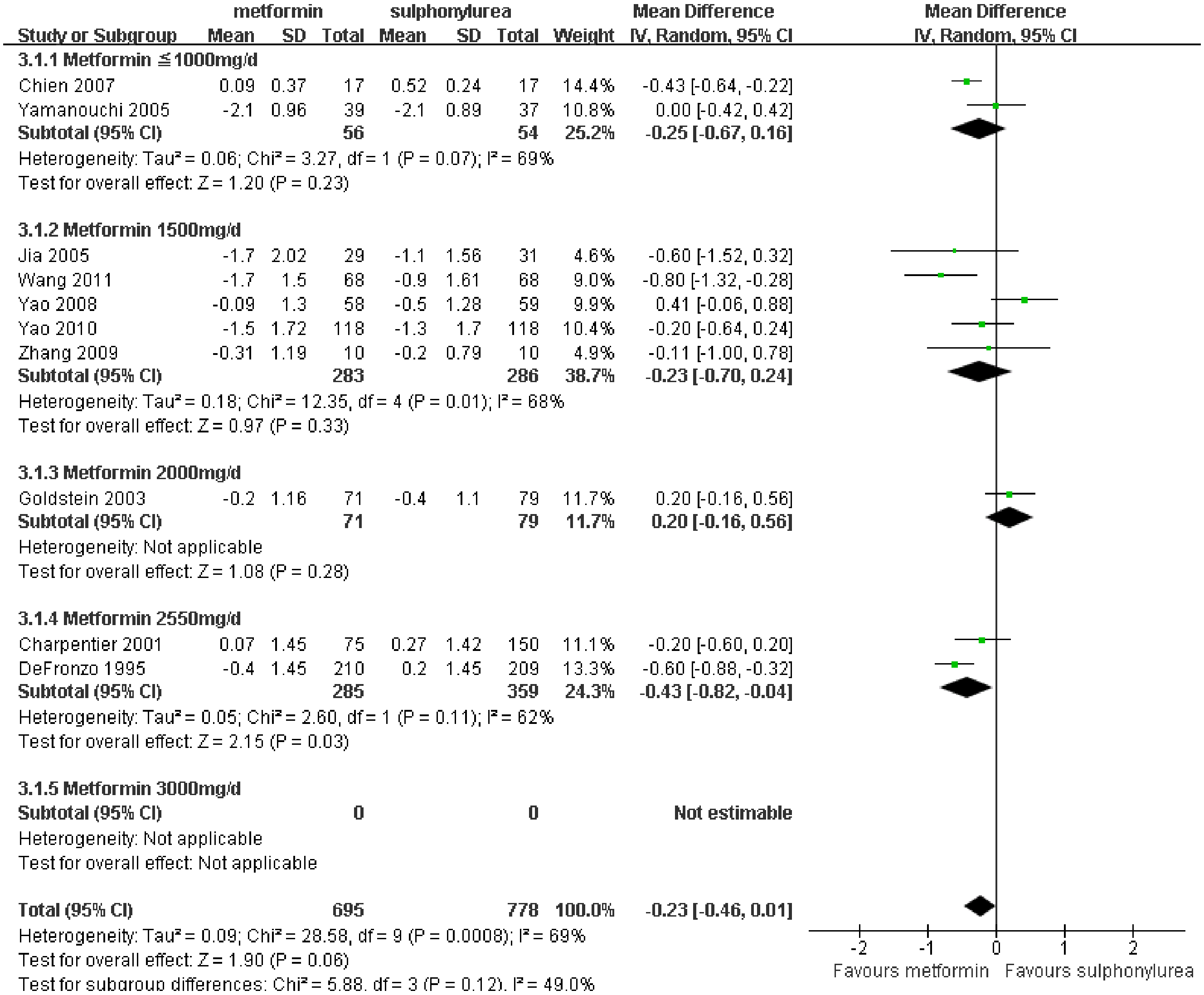

S9 Fig. Sensitivity analysis: Glucose lowering effect (reduction of HbA_{1c}) of metformin versus sulphonylureas (remove thirteen low-quality studies [14, 58, 60, 62, 64-66, 68, 73, 75-78] from the original data set)
