# Supplementary material for: Comparison of Glucose Lowering Effect of Metformin and Acarbose in Type 2 Diabetes Mellitus: A Meta-Analysis
Source: PLoS One. 2015 May 11;10(5):e0126704. doi: 10.1371/journal.pone.0126704 (PMC4427275; doi:10.1371/journal.pone.0126704)
Supplement: S10 Fig — (PDF) [file pone.0126704.s011.pdf]

Study or Subgroup	metformin Mean	SD	Total	sulphonylurea Mean	SD	Total	Weight	Mean Difference IV, Random, 95% CI
**3.1.2 Metformin 1500mg/d**								
Chen 2004	-1.69	1.94	32	-1.7	1.89	30	3.6%	0.01 [-0.94, 0.96]
Chen 2012	-1.3	0.8	26	-0.9	0.8	27	6.1%	-0.40 [-0.83, 0.03]
Jia 2005	-1.7	2.02	29	-1.1	1.56	31	3.7%	-0.60 [-1.52, 0.32]
Ling 2003	-2.96	0.94	44	-2.95	1.11	50	6.2%	-0.01 [-0.42, 0.40]
Liu 2013	-1.59	1.45	38	-1.28	0.55	35	5.8%	-0.31 [-0.81, 0.19]
Tang 2004	-0.5	1.48	29	-0.7	1.51	33	4.5%	0.20 [-0.55, 0.95]
Wang 2011	-1.7	1.5	68	-0.9	1.61	68	5.6%	-0.80 [-1.32, -0.28]
Xu 2006	-2.96	0.94	44	-3	1.11	50	6.2%	0.04 [-0.37, 0.45]
Yao 2008	-0.09	1.3	58	-0.5	1.28	59	5.9%	0.41 [-0.06, 0.88]
Yao 2010	-1.5	1.72	118	-1.3	1.7	118	6.1%	-0.20 [-0.64, 0.24]
Zhang 2009	-0.31	1.19	10	-0.2	0.79	10	3.8%	-0.11 [-1.00, 0.78]
**Subtotal (95% CI)**			**496**			**511**	**57.5%**	**-0.15 [-0.36, 0.06]**
Heterogeneity: Tau² = 0.05; Chi² = 16.32, df = 10 (P = 0.09); I² = 39%								
Test for overall effect: Z = 1.42 (P = 0.16)								
**3.1.3 Metformin 2000mg/d**								
Goldstein 2003	-0.2	1.16	71	-0.4	1.1	79	6.5%	0.20 [-0.16, 0.56]
Xuan 2012	-2.31	2.23	35	-3	3.02	35	2.6%	0.69 [-0.55, 1.93]
**Subtotal (95% CI)**			**106**			**114**	**9.1%**	**0.24 [-0.11, 0.59]**
Heterogeneity: Tau² = 0.00; Chi² = 0.55, df = 1 (P = 0.46); I² = 0%								
Test for overall effect: Z = 1.34 (P = 0.18)								
**3.1.4 Metformin 2550mg/d**								
Charpentier 2001	0.07	1.45	75	0.27	1.42	150	6.3%	-0.20 [-0.60, 0.20]
DeFronzo 1995	-0.4	1.45	210	0.2	1.45	209	6.9%	-0.60 [-0.88, -0.32]
Formoso 2008	-2.2	0.04	13	-1.4	0.03	13	7.5%	-0.80 [-0.83, -0.77]
Ning 2006	-2.02	2.13	50	-1.5	1.98	51	4.2%	-0.52 [-1.32, 0.28]
Tessier 1999	-1	1.56	18	-1	1.87	18	2.9%	0.00 [-1.13, 1.13]
**Subtotal (95% CI)**			**366**			**441**	**27.8%**	**-0.55 [-0.83, -0.27]**
Heterogeneity: Tau² = 0.05; Chi² = 12.93, df = 4 (P = 0.01); I² = 69%								
Test for overall effect: Z = 3.86 (P = 0.0001)								
**3.1.5 Metformin 3000mg/d**								
Lawrence 2004	-1.14	0.84	20	-1.21	0.84	20	5.6%	0.07 [-0.45, 0.59]
**Subtotal (95% CI)**			**20**			**20**	**5.6%**	**0.07 [-0.45, 0.59]**
Heterogeneity: Not applicable								
Test for overall effect: Z = 0.26 (P = 0.79)								
**Total (95% CI)**			**988**			**1086**	**100.0%**	**-0.19 [-0.44, 0.06]**
Heterogeneity: Tau² = 0.21; Chi² = 134.96, df = 18 (P < 0.00001); I² = 87%								
Test for overall effect: Z = 1.51 (P = 0.13)								
Test for subgroup differences: Chi² = 13.25, df = 3 (P = 0.004), I² = 77.4%								

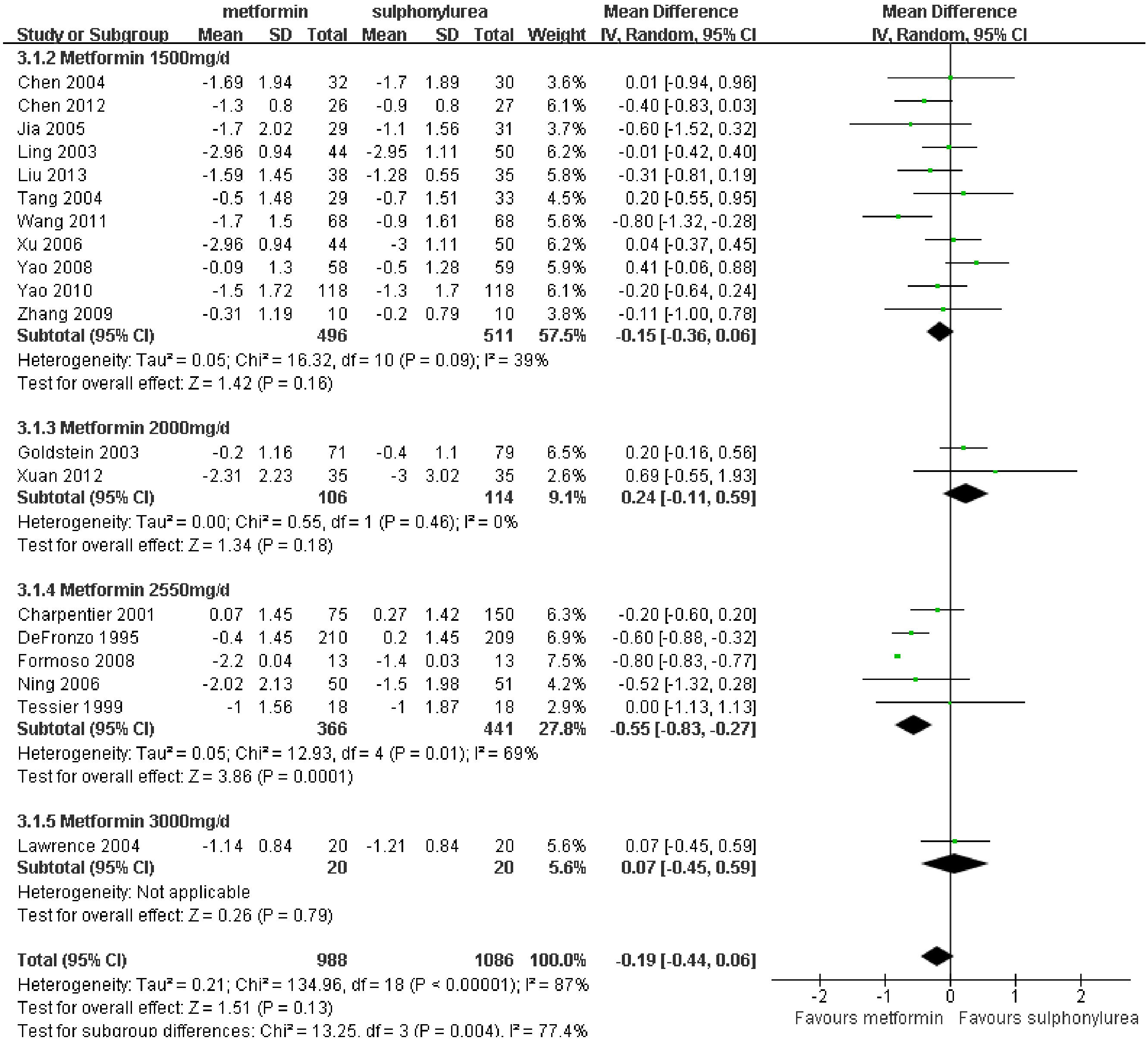

S10 Fig. Sensitivity analysis: Glucose lowering effect (reduction of HbA_{1c}) of metformin versus sulphonylureas (adjust drug doses, set ≥ 1500mg/d as maximum or maintenance dose of metformin)
