# Supplementary material for: Comparison of Glucose Lowering Effect of Metformin and Acarbose in Type 2 Diabetes Mellitus: A Meta-Analysis
Source: PLoS One. 2015 May 11;10(5):e0126704. doi: 10.1371/journal.pone.0126704 (PMC4427275; doi:10.1371/journal.pone.0126704)
Supplement: S11 Fig — (PDF) [file pone.0126704.s012.pdf]

	acarbose			sulphonylurea				Mean Difference
**Study or Subgroup**	**Mean**	**SD**	**Total**	**Mean**	**SD**	**Total**	**Weight**	**IV, Random, 95% CI**
**4.1.1 Western**								
Fischer 2003	0	1.63	25	-1.3	1.06	27	6.3%	1.30 [0.55, 2.05]
Hanefeld 2002	0.4	2.13	11	-0.8	1.62	8	1.7%	1.20 [-0.49, 2.89]
Rosenthal 2002	-0.5	1.43	32	-0.2	1.81	31	5.7%	-0.30 [-1.11, 0.51]
Salman 2001	-1.8	1.47	27	-2.2	0.86	30	8.0%	0.40 [-0.23, 1.03]
Van de Laar 2004	-1.1	1.68	32	-1.8	1.59	43	6.4%	0.70 [-0.05, 1.45]
**Subtotal (95% CI)**			**127**			**139**	**28.1%**	**0.59 [0.03, 1.15]**
Heterogeneity: Tau² = 0.22; Chi² = 8.99, df = 4 (P = 0.06); I² = 56%								
Test for overall effect: Z = 2.06 (P = 0.04)								
**4.1.2 Eastern**								
Chen 2004	-1.81	1.87	32	-1.73	1.89	30	4.6%	-0.08 [-1.02, 0.86]
Hasegawa 2008	-0.2	0.44	13	-0.3	0.39	13	15.1%	0.10 [-0.22, 0.42]
Hu 2012	-1.29	0.77	34	-1.48	0.81	34	13.5%	0.19 [-0.19, 0.57]
Li 2007	-0.94	1.98	30	-1.03	1.51	30	4.9%	0.09 [-0.80, 0.98]
Li 2008	-2.6	1.21	32	-2.53	1.27	30	8.2%	-0.07 [-0.69, 0.55]
Wang 1999	-1.2	1.92	32	-1.21	1.55	30	5.2%	0.01 [-0.86, 0.88]
Wang 2007	-2.7	0.93	70	-2.5	0.88	75	15.8%	-0.20 [-0.50, 0.10]
Yang 2010	-2.15	2.73	68	-1.96	2.77	66	4.6%	-0.19 [-1.12, 0.74]
**Subtotal (95% CI)**			**311**			**308**	**71.9%**	**-0.01 [-0.18, 0.16]**
Heterogeneity: Tau² = 0.00; Chi² = 3.39, df = 7 (P = 0.85); I² = 0%								
Test for overall effect: Z = 0.11 (P = 0.91)								
**Total (95% CI)**			**438**			**447**	**100.0%**	**0.16 [-0.07, 0.38]**
Heterogeneity: Tau² = 0.06; Chi² = 20.65, df = 12 (P = 0.06); I² = 42%								
Test for overall effect: Z = 1.37 (P = 0.17)								
Test for subgroup differences: Chi² = 4.04, df = 1 (P = 0.04), I² = 75.2%								

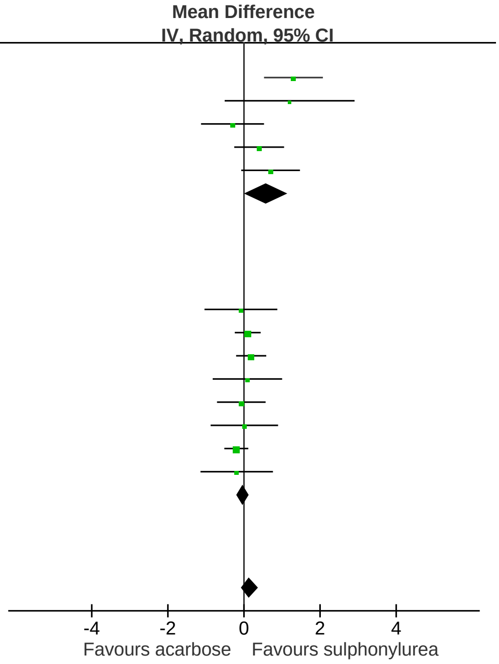

S11 Fig. Glucose lowering effect (reduction of HbA_{1c}) of acarbose versus sulphonylurea (Western and Eastern as subgroups)
