# Supplementary material for: Comparison of Glucose Lowering Effect of Metformin and Acarbose in Type 2 Diabetes Mellitus: A Meta-Analysis
Source: PLoS One. 2015 May 11;10(5):e0126704. doi: 10.1371/journal.pone.0126704 (PMC4427275; doi:10.1371/journal.pone.0126704)
Supplement: S12 Fig — (PDF) [file pone.0126704.s013.pdf]

Study or Subgroup	acarbose Mean	acarbose SD	acarbose Total	sulphonylurea Mean	sulphonylurea SD	sulphonylurea Total	Weight	Mean Difference IV, Fixed, 95% CI
**4.1.1 Acarbose 150mg/d**								
Chen 2004	-1.81	1.87	32	-1.73	1.89	30	2.6%	-0.08 [-1.02, 0.86]
**Subtotal (95% CI)**			**32**			**30**	**2.6%**	**-0.08 [-1.02, 0.86]**
Heterogeneity: Not applicable								
Test for overall effect: Z = 0.17 (P = 0.87)								
**4.1.2 Acarbose 300mg/d**								
Fischer 2003	0	1.63	25	-1.3	1.06	27	4.0%	1.30 [0.55, 2.05]
Hanefeld 2002	0.4	2.13	11	-0.8	1.62	8	0.8%	1.20 [-0.49, 2.89]
Hasegawa 2008	-0.2	0.44	13	-0.3	0.39	13	22.4%	0.10 [-0.22, 0.42]
Hu 2012	-1.29	0.77	34	-1.48	0.81	34	16.2%	0.19 [-0.19, 0.57]
Li 2007	-0.94	1.98	30	-1.03	1.51	30	2.9%	0.09 [-0.80, 0.98]
Li 2008	-2.6	1.21	32	-2.53	1.27	30	6.0%	-0.07 [-0.69, 0.55]
Rosenthal 2002	-0.5	1.43	32	-0.2	1.81	31	3.5%	-0.30 [-1.11, 0.51]
Salman 2001	-1.8	1.47	27	-2.2	0.86	30	5.7%	0.40 [-0.23, 1.03]
Van de Laar 2004	-1.1	1.68	32	-1.8	1.59	43	4.0%	0.70 [-0.05, 1.45]
Wang 1999	-1.2	1.92	32	-1.21	1.55	30	3.0%	0.01 [-0.86, 0.88]
Wang 2007	-2.7	0.93	70	-2.5	0.88	75	26.2%	-0.20 [-0.50, 0.10]
Yang 2010	-2.15	2.73	68	-1.96	2.77	66	2.6%	-0.19 [-1.12, 0.74]
**Subtotal (95% CI)**			**406**			**417**	**97.4%**	**0.10 [-0.05, 0.25]**
Heterogeneity: Chi² = 20.52, df = 11 (P = 0.04); I² = 46%								
Test for overall effect: Z = 1.27 (P = 0.20)								
**Total (95% CI)**			**438**			**447**	**100.0%**	**0.09 [-0.06, 0.25]**
Heterogeneity: Chi² = 20.65, df = 12 (P = 0.06); I² = 42%								
Test for overall effect: Z = 1.23 (P = 0.22)								
Test for subgroup differences: Chi² = 0.14, df = 1 (P = 0.71), I² = 0%								

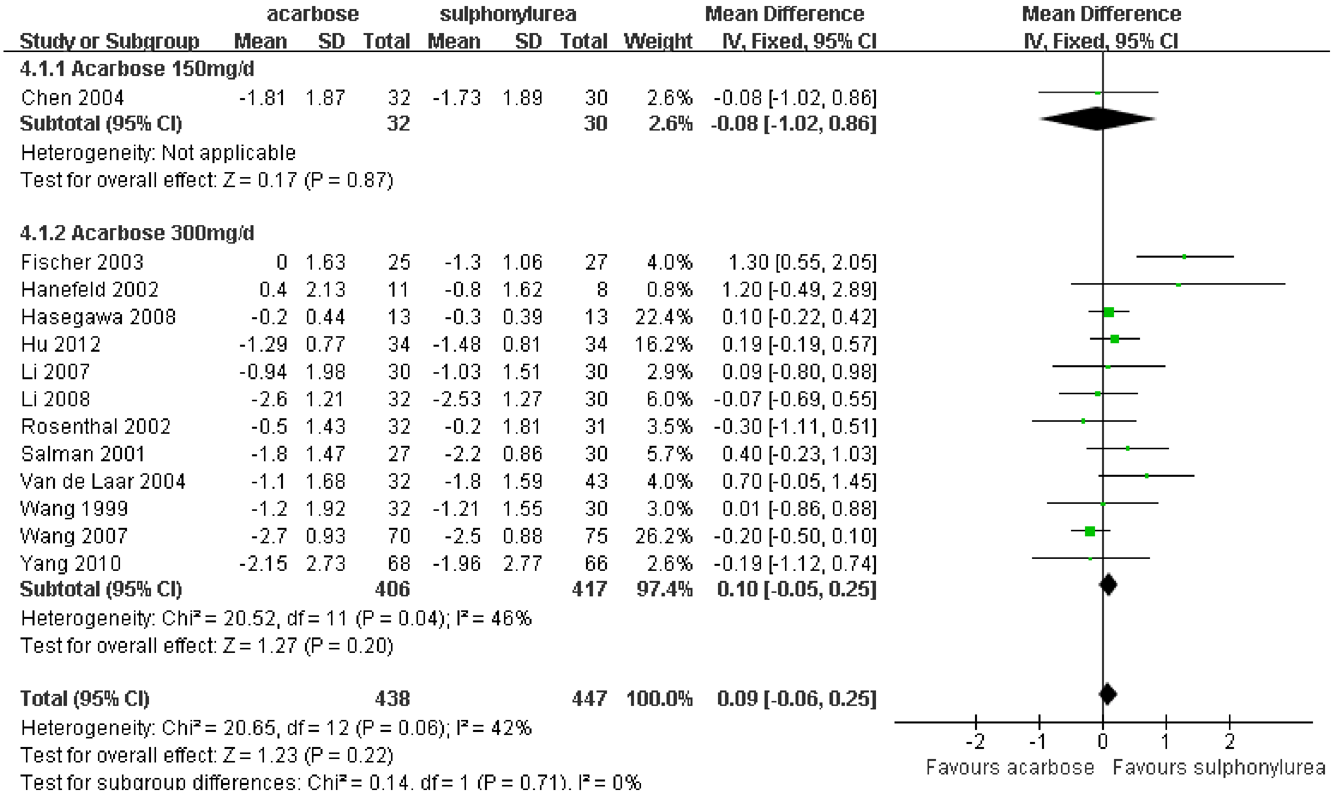

S12 Fig. Sensitivity analysis: Glucose lowering effect (reduction of HbA_{1c}) of acarbose versus sulphonylureas (use a fixed-effect model instead)
