# Supplementary material for: Comparison of Glucose Lowering Effect of Metformin and Acarbose in Type 2 Diabetes Mellitus: A Meta-Analysis
Source: PLoS One. 2015 May 11;10(5):e0126704. doi: 10.1371/journal.pone.0126704 (PMC4427275; doi:10.1371/journal.pone.0126704)
Supplement: S13 Fig — (PDF) [file pone.0126704.s014.pdf]

Study or Subgroup	acarbose Mean	acarbose SD	acarbose Total	sulphonylurea Mean	sulphonylurea SD	sulphonylurea Total	Weight	Mean Difference IV, Random, 95% CI
**4.1.2 Acarbose 300mg/d**								
Fischer 2003	0	1.63	25	-1.3	1.06	27	6.8%	1.30 [0.55, 2.05]
Hanefeld 2002	0.4	2.13	11	-0.8	1.62	8	1.8%	1.20 [-0.49, 2.89]
Hasegawa 2008	-0.2	0.44	13	-0.3	0.39	13	15.3%	0.10 [-0.22, 0.42]
Hu 2012	-1.29	0.77	34	-1.48	0.81	34	13.9%	0.19 [-0.19, 0.57]
Li 2007	-0.94	1.98	30	-1.03	1.51	30	5.4%	0.09 [-0.80, 0.98]
Li 2008	-2.6	1.21	32	-2.53	1.27	30	8.7%	-0.07 [-0.69, 0.55]
Rosenthal 2002	-0.5	1.43	32	-0.2	1.81	31	6.2%	-0.30 [-1.11, 0.51]
Salman 2001	-1.8	1.47	27	-2.2	0.86	30	8.5%	0.40 [-0.23, 1.03]
Van de Laar 2004	-1.1	1.68	32	-1.8	1.59	43	6.8%	0.70 [-0.05, 1.45]
Wang 1999	-1.2	1.92	32	-1.21	1.55	30	5.6%	0.01 [-0.86, 0.88]
Wang 2007	-2.7	0.93	70	-2.5	0.88	75	16.0%	-0.20 [-0.50, 0.10]
Yang 2010	-2.15	2.73	68	-1.96	2.77	66	5.0%	-0.19 [-1.12, 0.74]
**Subtotal (95% CI)**			**406**			**417**	**100.0%**	**0.17 [-0.07, 0.41]**
Heterogeneity: Tau² = 0.07; Chi² = 20.52, df = 11 (P = 0.04); I² = 46%								
Test for overall effect: Z = 1.42 (P = 0.16)								
**Total (95% CI)**			**406**			**417**	**100.0%**	**0.17 [-0.07, 0.41]**
Heterogeneity: Tau² = 0.07; Chi² = 20.52, df = 11 (P = 0.04); I² = 46%								
Test for overall effect: Z = 1.42 (P = 0.16)								
Test for subgroup differences: Not applicable								

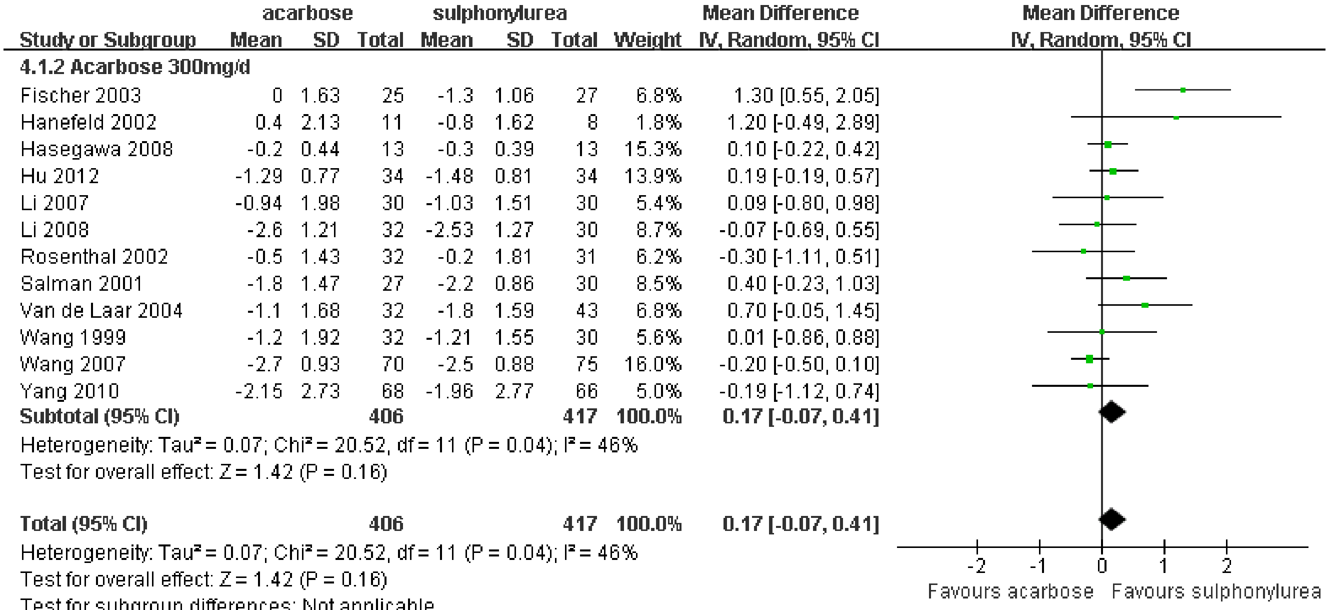

S13 Fig. Sensitivity analysis: Glucose lowering effect (reduction of HbA_{1c}) of acarbose versus sulphonylureas (adjust drug doses, set ≥ 300 mg/d as maximum or maintenance dose of acarbose)
