# Supplementary material for: Comparison of Glucose Lowering Effect of Metformin and Acarbose in Type 2 Diabetes Mellitus: A Meta-Analysis
Source: PLoS One. 2015 May 11;10(5):e0126704. doi: 10.1371/journal.pone.0126704 (PMC4427275; doi:10.1371/journal.pone.0126704)
Supplement: S1 Table — (DOC) [file pone.0126704.s015.doc]

**S1 Table.** **Search strategy**

| **Keywords** | **Search Steps** |
| --- | --- |
| Type 2 diabetes mellitus | |
| 1. | Diabetes mellitus, non-insulin-dependent [MeSH, all subheadings included] |
| 2. | NIDDM |
| 3. | "Non-insulin-dependent" or "Noninsulin-dependent" or "Non insulin dependent" |
| 4. | "Type II diabet*" or "Type 2 diabet*" |
| 5. | #1 or #2 or #3 or #4 |
| Metformin |  |
| 6. | Biguanides [MeSH, all subheadings included] |
| 7. | Biguanides* |
| 8. | Metformin [MeSH, all subheadings included] |
| 9. | Glucophag* |
| 10. | Metformin* |
| 11. | #6 or #7 or #8 or #9 or #10 |
| Acarbose |  |
| 12. | Acarbose [MeSH, all subheadings included] |
| 13. | acarbose OR (alph* glucos* inh*) OR (alf* glucos* inh*) OR glucobay OR precos* OR prandas* OR akarbos* |
| 14. | #12 or #13 |
| Metformin or acarbose | |
| 15. | #11 or #14 |
| Type 2 diabetes mellitus and ( metformin or acarbose ) | |
| 16. | #5 and #15 |
| Filters activated | |
| 17. | Clinical Trial, Humans. |
