# Supplementary material for: Comparison of Glucose Lowering Effect of Metformin and Acarbose in Type 2 Diabetes Mellitus: A Meta-Analysis
Source: PLoS One. 2015 May 11;10(5):e0126704. doi: 10.1371/journal.pone.0126704 (PMC4427275; doi:10.1371/journal.pone.0126704)
Supplement: S2 Table — (DOC) [file pone.0126704.s016.doc]

**S2 Table. Characteristics of Studies included in the indirect comparisons**

| **Study** | **Location** | **Trial Group** | | | | | **Control Group** | | | | | **Intervention characteristics** | | |
| --- | --- | --- | --- | --- | --- | --- | --- | --- | --- | --- | --- | --- | --- | --- |
|  |  | **Size** | **Age (y)** | **DM Duration**  **(mo/y)** | **BMI (kg/m2)** | **Drug** | **Size** | **Age (y)** | **DM Duration**  **(mo/y)** | **BMI (kg/m2)** | **Drug** | **Trial Group dose (mg/d)** | **Basic treatment** | **Duration**  **(wk)** |
| Goldstein 2007 [22] | US | 178 | 18~78 | NR | NR | MET | 165 | 18~78 | NR | NR | PLA | 1000 | Diet+exercise | 24 |
| Haak 2012 [23] | Germany, UK | 141 | 52.9(10.4) | NR | 28.9(4.8) | MET | 65 | 55.7(11.0) | NR | 28.6(5.2) | PLA | 1000 | Null | 24 |
| Mather 2001 [24] | US, Canada | 28 | 50.7(1.8) | NR | 32.1(1.3) | MET | 15 | 54.8(2.6) | NR | 33.1(1.9) | PLA | 1000 | diet | 12 |
| Uehara 2001 [25] | Brazil | 11 | 57.2(4.3) | 36.6(23.8)mo | 29.2(2.1) | MET | 11 | 57.5(6.7) | 45.5(43.1)mo | 30.1(2.5) | PLA | 1000 | Diet+exercise | 12 |
| Wu 2001 [26] | China | 60 | 49.8(6.7) | 4.2(2.1)y | 26.89(0.39) | MET | 60 | 50.4(6.4) | 4.3(2.4)y | 27.42(0.32) | PLA | 1000 | Diet+exercise | 24 |
| Chiasson 2001 [27] | Canada, France | 81 | 57.9(8.6) | 7.5 (7.4)y | 30.7(5.1) | MET | 82 | 57.7(9.9) | 5.1(4.9)y | 31.1(4.4) | PLA | 1500 | Diet+exercise | 36 |
| Horton 2004 [28] | US | 98 | 55.4(1.1) | 3.7 (0.4)y | 29.9(0.4) | MET | 98 | 59.0(1.1) | 4.2(0.4)y | 29.5(0.4) | PLA | 1500 | Diet+exercise | 24 |
| List 2009 [29] | US, Canada | 51 | 54 (9) | ND | 32(5) | MET | 44 | 53(11) | ND | 32(5) | PLA | 1500 | Diet+exercise | 12 |
| Chakraborty 2011 [30] | India | 110 | 30~55 | 1~5y | 27(2.4) | MET | 98 | 30~55 | 1~5y | 27(1.3) | PLA | 1700 | Diet+exercise | 24 |
| Lee 1998 [31] | US | 24 | 59(3) | 4 (1)y | 40.0(1.8) | MET | 24 | 61(2) | 3(2)y | 39.6(1.2) | PLA | 1700 | Diet | 24 |
| Manzella 2004 [32] | Italy | 60 | 57(11) | NR | 29.5(0.1) | MET | 60 | 57(11) | NR | 29.2(0.2) | PLA | 1700 | Diet | 17 |
| Pirags 2012 [33] | Latvia | 33 | 60 | NR | 32.7 (0.78) | MET | 33 | 58.9 | NR | 32.0 (0.75) | PLA | 1700 | Diet | 8 |
| Goldstein 2007 [22] | US | 177 | 18~78 | NR | NR | MET | 165 | 18~78 | NR | NR | PLA | 2000 | Diet+exercise | 24 |
| Haak 2012 [23] | Germany, UK | 138 | 55.2( 10.6) | NR | 29.5(5.3) | MET | 65 | 55.7( 11.0) | NR | 28.6( 5.2) | PLA | 2000 | Null | 24 |

**S2** Table. Continued

| **Study** | **Location** | **Trial Group** | | | | | **Control Group** | | | | | **Intervention characteristics** | | |
| --- | --- | --- | --- | --- | --- | --- | --- | --- | --- | --- | --- | --- | --- | --- |
|  |  | **Size** | **Age (y)** | **DM Duration**  **(mo/y)** | **BMI (kg/m2)** | **Drug** | **Size** | **Age (y)** | **DM Duration**  **(mo/y)** | **BMI (kg/m2)** | **Drug** | **Trial Group dose (mg/d)** | **Basic treatment** | **Duration**  **(wk)** |
| Hällsten 2002 [34] | Finland | 13 | 57.8(2.2) | NR | 29.9(1.1) | MET | 14 | 57.7(1.9) | NR | 30.3(1.2) | PLA | 2000 | Diet | 26 |
| Hällsten 2004 [35] | Finland | 9 | 54.0(1.4) | ND | 29.9(1.1) | MET | 11 | 57.5(2.4) | ND | 30.6(1.5) | PLA | 2000 | Diet | 26 |
| Karlsson 2005 [36] | Finland | 9 | 57.7(2.9) | ND | 28.8(1.3) | MET | 11 | 58.6(2.5) | ND | 29.4(1.3) | PLA | 2000 | Diet+exercise | 26 |
| Viljanen 2005 [37] | Finland | 12 | 57.8(8.7) | NR | 29.6(4.0) | MET | 11 | 58.7(8.3) | NR | 29.8(4.1) | PLA | 2000 | Diet | 26 |
| DeFronzo 1995 [38] | US | 143 | 53(1) | 6 (0.5)y | 29.9(0.3) | MET | 146 | 53(1) | 6(0.6)y | 29.2(0.3) | PLA | 2550 | Diet | 29 |
| Del Prato 2003 [39] | France, Italy, Netherlands | 250 | 56(9) | NR | 29.7(4.2) | MET | 127 | 56(9) | NR | 29.9(3.9) | PLA | 2550 | Diet | 29 |
| Damsbo 1998 [40] | Denmark | 9 | 51(40-60) | ND | 31.6(1.2) | MET | 9 | 53(40-63) | ND | 31.9(1.5) | PLA | 3000 | Diet | 12 |
| Dornan 1991 [41] | UK | 30 | 55(1) | NR | 30(1) | MET | 30 | 55(1) | NR | 30(1) | PLA | 3000 | Diet | 32 |
| Fischer 1998 [42] | Europe | 86 | 58.5(8.4) | 26mo | 27.3(3.5) | AK | 81 | 52.7(8.7) | 24mo | 26.9(2.9) | PLA | 75 | Diet | 24 |
| Fischer 1998 [42] | Europe | 88 | 55.5(9.6) | 20mo | 27.6(3.5) | AK | 81 | 52.7(8.7) | 24mo | 26.9(2.9) | PLA | 150 | Diet | 24 |
| Chan 1998 [43] | Asia | 59 | 52.8(10.2) | 2.7(3.5)y | 25.4(3.9) | AK | 62 | 54.0(10.0) | 2.1(3.4)y | 25.6(3.8) | PLA | 300 | Diet | 24 |
| Derosa 2011 [44] | Italy | 88 | 56(7) | 4.3(1.3)mo | 26.6(0.8) | AK | 87 | 56(7) | 4.3(1.3)mo | 26.8(0.9) | PLA | 300 | Diet+exercise | 30 |
| Fischer 1998 [42] | Europe | 78 | 56.8(9.4) | 17mo | 27.6(3.7) | AK | 81 | 52.7(8.7) | 24mo | 26.9(2.9) | PLA | 300 | Diet | 24 |
| Fischer 2003 [45] | Germany | 25 | 59.4(5.6) | 94(11.9)mo | 27.3(0.8) | AK | 25 | 58.6(6.3) | 77.3(10.7)mo | 27(0.7) | PLA | 300 | Diet | 16 |

**S2** Table. Continued

| **Study** | **Location** | **Trial Group** | | | | | **Control Group** | | | | | **Intervention characteristics** | | |
| --- | --- | --- | --- | --- | --- | --- | --- | --- | --- | --- | --- | --- | --- | --- |
|  |  | **Size** | **Age (y)** | **DM Duration**  **(mo/y)** | **BMI (kg/m2)** | **Drug** | **Size** | **Age (y)** | **DM Duration**  **(mo/y)** | **BMI (kg/m2)** | **Drug** | **Trial Group dose (mg/d)** | **Basic treatment** | **Duration (wk)** |
| Hanefeld 1991 [46] | Germany | 47 | 43~70 | 8~235mo | 20.6~36.1 | AK | 47 | 43~70 | 5~197mo | 19.4~37.0 | PLA | 300 | Diet | 24 |
| Hanefeld 2002 [47] | Germany | 11 | 60.4(1.3) | 92.4(16.2)y | 27.5(0.7) | AK | 8 | 59(1.6) | 81.2(19.2)mo | 27.2(1.1) | PLA | 300 | Diet | 16 |
| Hotta 1993 [48] | Japan | 19 | 49.8 | 4.6y | 23.5 | AK | 18 | 47.9 | 4.8y | 22.9 | PLA | 300 | Diet | 24 |
| Hwu 2003 [49] | Asia | 54 | 58.1(8.4) | 160.7(109.3)mo | 24.2(3.5) | AK | 53 | 54.7(8.6) | 131(73.6)mo | 23.9(3.7) | PLA | 300 | Diet | 18 |
| Josse 2003 [50] | Canada | 93 | 69.7(0.5) | 5.8(0.7) | 28.3(0.4) | AK | 99 | 70.3(0.5) | 4.8(0.5)y | 28.6(0.4) | PLA | 300 | Diet | 52 |
| Kirkman 2006 [51] | US | 81 | 53.7(11.0) | NR | 35.1(7.2) | AK | 76 | 53.7(11.7) | NR | 35.2(7.1) | PLA | 300 | Diet | 52 |
| Meneilly 2000 [52] | Canada | 22 | 68(1) | NR | 28(1) | AK | 23 | 70(1) | NR | 29(1) | PLA | 300 | Diet | 52 |
| Rosenbaum 2002 [53] | Brazil | 20 | 59.8(8.2) | 82mo | 30.3(2.9) | AK | 20 | 62(9.7) | 81mo | 31.7(3.9) | PLA | 300 | Diet+exercise | 24 |
| Scott 1999 [54] | Australia, New Zealand | 41 | 56(9) | 21(15)mo | 31(3) | AK | 42 | 57(8) | 26(17)mo | 29(3) | PLA | 300 | Diet | 16 |
| Wolever 1997 [55] | Canada | 25 | 57.2(1.1) | 5.2(0.6)y | 28.8(0.5) | AK | 29 | 57.2(1.1) | 5.2(0.6)y | 28.8(0.5) | PLA | 300 | Diet | 52 |
| Wu 2003 [56] | China | 80 | 50(7) | 4.2(2.1)y | 26.5(2.3) | AK | 80 | 50(6) | 4.3(2.4)y | 27.2(2.5) | PLA | 300 | Diet+exercise | 24 |
| Buchanan 1988 [57] | UK | 9 | 60.1(6.8) | 44.9(28.6)mo | NR | AK | 11 | 57.6(8.2) | 50.6(30.1)mo | NR | PLA | 400 | Diet | 16 |
| Fischer 1998 [42] | Europe | 87 | 59.4(8.6) | 21mo | 27.2(3.3) | AK | 81 | 52.7(8.7) | 24mo | 26.9(2.9) | PLA | 600 | Diet | 24 |

**S2** Table. Continued

|  | **Location** | **Trial Group** | | | | | **Control Group** | | | | | **Intervention characteristics** | | |
| --- | --- | --- | --- | --- | --- | --- | --- | --- | --- | --- | --- | --- | --- | --- |
| **Study** |  | **Size** | **Age (y)** | **DM Duration**  **(mo/y)** | **BMI (kg/m2)** | **Drug** | **Size** | **Age (y)** | **DM Duration**  **(mo/y)** | **BMI (kg/m2)** | **Drug** | **Trial Group dose (mg/d)** | **Basic treatment** | **Duration**  **(wk)** |
| Amador-Licona 2000 [58] | Mexico | 28 | 49.3(8.2) | 4.5(1-14)y | 22.8~44.2 | MET | 23 | 48.2(7) | 4(1-15)y | 23~44.8 | SUs | 850 | Diet | 12 |
| Chien 2007 [59] | Taiwan | 17 | 59(9) | 6.4(4.8)y | 25.7(3.2) | MET | 17 | 63(7) | 8.6(5.9)y | 25.3(34) | SUs | 1000 | Diet | 16 |
| Ramachandran 2004 [60] | India | 21 | 44.4(10.6) | ND | 25.7(2.6) | MET | 18 | 45.3(10.3) | ND | 24.6(2.5) | SUs | 850 | Diet | 14 |
| Yamanouchi 2005 [61] | Japan | 39 | 54.7(9.8) | 3.0(2.5)y | ≥25 | MET | 37 | 55.6(9.3) | 3.3(2.6)y | ≥25 | SUs | 750 | Diet +exercise | 52 |
| Chen 2004 [14] | China | 32 | 20~44 | ND | 25.19(0.53) | MET | 30 | 20~44 | ND | 24.99(0.52) | SUs | 1500 | Null | 36 |
| Chen 2012 [62] | China | 26 | 48.55(7.18) | ≤ 3mo | NR | MET | 27 | 48.34(7.21) | ≤ 3mo | NR | SUs | 1500 | Diet +exercise | 52 |
| Jia 2005 [63] | China | 29 | 35~65 | NR | NR | MET | 31 | 35~65 | NR | NR | SUs | 1500 | Null | 12 |
| Ling 2003 [64] | China | 44 | 53(9.2) | NR | NR | MET | 50 | 54(8.3) | NR | NR | SUs | 1500 | Null | 12 |
| Liu 2013 [65] | China | 38 | 53.1(8.3) | NR | 26.2(3.5) | MET | 35 | 57.4(6.9) | NR | 24.8(1.9) | SUs | 1500 | Diet | 14 |
| Tang 2004 [66] | China | 29 | 53.8(9.7) | 0~5y | 24.6(2.2) | MET | 33 | 56.4(8.8) | 0~5y | 23.3(1.7) | SUs | 1500 | Diet +exercise | 26 |
| Wang 2011 [67] | China | 68 | 48.53(8.74) | ND | 28.20(3.70) | MET | 68 | 49.47(8.52) | ND | 28.60(4.10) | SUs | 1500 | Diet +exercise | 12 |
| Xu 2006 [68] | China | 44 | 54(9.2) | NR | NR | MET | 50 | 54(8.3) | NR | NR | SUs | 1500 | Null | 12 |
| Yao 2008 [69] | China | 58 | 56.83(10.02) | 5.21(4.30)y | 25.38(2.98) | MET | 59 | 57.59(9.07) | 4.75(3.92)y | 25.93 (3.13 | SUs | 1500 | Null | 12 |
| Yao 2010 [70] | China | 118 | 30~75 | NR | ≤ 32 | MET | 118 | 30~75 | NR | ≤ 32 | SUs | 1500 | Null | 12 |

**S2** Table. Continued

|  | **Location** | **Trial Group** | | | | | **Control Group** | | | | | **Intervention characteristics** | | |
| --- | --- | --- | --- | --- | --- | --- | --- | --- | --- | --- | --- | --- | --- | --- |
| **Study** |  | **Size** | **Age (y)** | **DM Duration**  **(mo/y)** | **BMI (kg/m2)** | **Drug** | **Size** | **Age (y)** | **DM Duration**  **(mo/y)** | **BMI (kg/m2)** | **Drug** | **Trial Group dose (mg/d)** | **Basic treatment** | **Duration**  **(wk)** |
| Zhang 2009 [71] | China | 10 | 49.8(7.1) | 1.26(1.03)y | 25.36(1.66) | MET | 10 | 54(5.14) | 2.5(2.32)y | 24.44(1.89) | SUs | 1500 | Null | 12 |
| Goldstein 2003 [72] | US | 71 | 56.6(9.7) | 7.3(4.9)y | 31.6(4.3) | MET | 79 | 57.4(9.2) | 6.5(4.4)y | 30.6(4.8) | SUs | 2000 | Null | 18 |
| Xuan 2012 [73] | China | 35 | 35~70 | NR | 19~35 | MET | 35 | 35~70 | NR | 19~35 | SUs | 2000 | Diet+exercise | 12 |
| Charpentier 2001 [74] | France | 75 | 56.7 | <1y | 29.2 | MET | 150 | 55.4 | <1y | 29.3 | SUs | 2550 | Diet | 20 |
| DeFronzo 1995 [38] | US | 210 | 55(1) | 8.4(0.4)y | 29.4(0.3) | MET | 209 | 56(1) | 8.7(0.4)y | 29.1(0.3) | SUs | 2550 | Diet | 29 |
| Formoso 2008 [75] | Italy | 13 | 58.8(1.32) | 6mo | 33.4(1.22) | MET | 13 | 57.2(1.56) | 6mo | 30.9(1.05) | SUs | 2550 | Diet | 12 |
| Ning 2006 [76] | China | 50 | 52.66(9.62) | >1 | 25.91(2.19) | MET | 51 | 56.39(8.16) | >1y | 23.63(2.51) | SUs | 2550 | Null | 52 |
| Tessier 1999 [77] | Canada | 18 | 59.1(7.1) | 5.4(6.5)y | 29.3(3) | MET | 18 | 59.3(7.3) | 4.7(6.1)y | 28.6(4) | SUs | 2550 | Diet | 24 |
| Lawrence 2004 [78] | UK | 20 | 59.5(9.3) | NR | >27 | MET | 20 | 63.5(11.4) | NR | >27 | SUs | 3000 | Diet | 12 |
| Chen 2004 [14] | China | 32 | 20~44 | ND | 25.19(0.53) | AK | 30 | 20~44 | ND | 24.99(0.52) | SUs | 150 | Null | 36 |
| Fischer 2003 [45] | Germany | 25 | 59.4(5.6) | 94(11.9)mo | 27.3(0.8) | AK | 27 | 58.1(7) | 69.5(9.6)mo | 27.7(0.6) | SUs | 300 | Diet | 16 |
| Hanefeld 2002 [47] | Germany | 11 | 60.4(1.3) | 92.4(16.2)y | 27.5(0.7) | AK | 8 | 60.6(2.5) | 85.4(14.7)mo | 27.1(1.1) | SUs | 300 | Diet | 16 |
| Hasegawa 2008 [79] | Japan | 13 | 56.3(6.5) | 7.2(4.2)mo | 23.4(3.3) | AK | 13 | 55.6(7.0) | 7.1(5.1)mo | 23.5(3.3) | SUs | 300 | Diet | 12 |
| Hu 2012 [80] | China | 34 | 49.34(6.07) | ND | 26.98(1.72) | AK | 34 | 49.44(6.32) | ND | 27.06(2.33) | SUs | 300 | Diet+exercise | 24 |

**S2** Table. Continued

|  | **Location** | **Trial Group** | | | | | **Control Group** | | | | | **Intervention characteristics** | | |
| --- | --- | --- | --- | --- | --- | --- | --- | --- | --- | --- | --- | --- | --- | --- |
| **Study** |  | **Size** | **Age (y)** | **DM Duration**  **(mo/y)** | **BMI (kg/m2)** | **Drug** | **Size** | **Age (y)** | **DM Duration**  **(mo/y)** | **BMI (kg/m2)** | **Drug** | **Trial Group dose (mg/d)** | **Basic treatment** | **Duration**  **(wk)** |
| Li 2007 [81] | China | 30 | >60 | NR | NR | AK | 30 | >60 | NR | NR | SUs | 300 | Null | 24 |
| Li 2008 [82] | China | 32 | 54.3(1.6) | ND | NR | AK | 30 | 54.3(1.6) | ND | NR | SUs | 300 | Diet+exercise | 12 |
| Rosenthal 2002 [83] | Germany | 32 | 57.4(8.6) | NR | 29.1(4.3) | AK | 31 | 57.7(10.5) | NR | 28.8(4.3) | SUs | 300 | Null | 24 |
| Salman 2001 [84] | Turkey (Istanbul) | 27 | 52.6(9.1) | 4.2(3.4)y | 30.2(3.8) | AK | 30 | 56.1(8.7) | 4.7(5.6)y | 29.2(2.8) | SUs | 300 | Diet | 24 |
| Van de Laar 2004 [85] | Netherlands | 32 | 40~70 | NR | 29.1(4.6) | AK | 43 | 40~70 | NR | 28.8(5.5) | SUs | 300 | Diet | 30 |
| Wang 1999 [86] | China | 32 | 65.93(6.25) | NR | 26.85(3.89) | AK | 30 | 64.61(8.07) | NR | 26.02(2.58) | SUs | 300 | Diet+exercise | 17 |
| Wang 2007 [87] | China | 70 | 53(7.8) | NR | NR | AK | 75 | 53(8.5) | NR | NR | SUs | 300 | Null | 12 |
| Yang 2010 [88] | China | 68 | 50.8(7.9) | 8.2(3.4)y | NR | AK | 66 | 52.1(8.7) | 7.9(3.8)y | NR | SUs | 300 | Diet+exercise | 12 |

Data are expressed as n, median (minimum-maximum), mean (SD); Size, sample size; DM, type 2 diabetes; BMI, body mass index; y, year; mo, month; mo/y, month or year; wk, week; US, United States; UK, United Kingdom, ND, newly diagnosed; MET, metformin; PLA, placebo; AK, acarbose; SUs, sulphonylureas; NR, not reported; Null, with no basic treatment; In metformin vs. placebo (sulphonylureas) group, trial group is metformin, trial intervention is daily dose of metformin; In acarbose vs. placebo (sulphonylureas) group, trial group is acarbose, trial intervention is daily dose of acarbose.
