# Supplementary material for: Comparison of Glucose Lowering Effect of Metformin and Acarbose in Type 2 Diabetes Mellitus: A Meta-Analysis
Source: PLoS One. 2015 May 11;10(5):e0126704. doi: 10.1371/journal.pone.0126704 (PMC4427275; doi:10.1371/journal.pone.0126704)
Supplement: S3 Table — (DOC) [file pone.0126704.s017.doc]

**S3 T**able. Quality of Studies included in the meta-analyses.

| **Study** | **Selection Bias** | | **Performance Bias** | **Attrition Bias** | **Overall Quality** |
| --- | --- | --- | --- | --- | --- |
|  | **Randomization** | **Allocation Concealment** | **Blinding** | **Dropout/**  **loss to Follow-up** |  |
| Chen 2004 [14] | B | B | C | B | C |
| Chou 2013 [15] | A | B | C | A | C |
| Hong 2011 [16] | B | B | C | B | C |
| Tang 2013 [17] | B | B | C | A | C |
| Wang 2005 [18] | B | B | C | B | C |
| Yang 2009 [19] | B | B | B | A | C |
| Zhang 2011 [20] | B | B | C | A | C |
| Zhu 2011 [21] | B | B | C | A | C |
| Goldstein 2007 [22] | A | A | A | A | A |
| Haak 2012 [23] | A | B | A | A | B |
| Mather 2001 [24] | A | A | A | A | A |
| Uehara 2001 [25] | A | B | A | A | B |
| Wu 2001 [26] | A | A | A | B | B |
| Chiasson 2001 [27] | B | B | A | A | B |
| Horton 2004 [28] | A | B | A | A | B |
| List 2009 [29] | B | B | A | A | B |
| Chakraborty 2011 [30] | A | B | A | B | B |
| Lee 1998 [31] | A | A | A | A | A |
| Manzella 2004 [32] | B | B | A | A | B |
| Pirags 2012 [33] | A | B | B | A | B |
| Hällsten 2002 [34] | B | B | B | A | C |
| Hällsten 2004 [35] | A | B | A | B | B |
| Karlsson 2005 [36] | A | A | A | B | B |
| Viljanen 2005 [37] | A | B | A | B | B |
| DeFronzo 1995 [38] | A | B | A | B | B |
| Del Prato 2003 [39] | B | A | A | B | B |
| Damsbo 1998 [40] | A | B | A | A | B |
| Dornan 1991 [41] | B | B | A | A | B |
| Fischer 1998 [42] | A | A | A | A | A |
| Chan 1998 [43] | B | B | A | A | B |
| Derosa 2011 [44] | A | A | A | A | A |
| Fischer 2003 [45] | B | B | B | B | C |
| Hanefeld 1991 [46] | B | B | B | A | B |
| Hanefeld 2002 [47] | B | B | B | B | C |

**S3 Table. Continued**

| **Study** | **Selection Bias** | | **Performance Bias** | **Attrition Bias** | **Overall Quality** |
| --- | --- | --- | --- | --- | --- |
|  | **Randomization** | **Allocation Concealment** | **Blinding** | **Dropout/loss to Follow-up** |  |
| Hotta 1993 [48] | B | A | A | B | B |
| Hwu 2003 [49] | B | B | A | A | B |
| Josse 2003 [50] | B | B | A | A | B |
| Kirkman 2006 [51] | A | B | A | B | B |
| Meneilly 2000 [52] | B | B | B | B | C |
| Rosenbaum 2002 [53] | B | B | B | A | C |
| Scott 1999 [54] | B | B | A | A | B |
| Wolever 1997 [55] | A | B | A | B | B |
| Wu 2003 [56] | A | A | A | B | B |
| Buchanan 1988 [57] | B | A | A | B | B |
| Amador-Licona 2000 [58] | B | B | C | A | C |
| Chien 2007 [59] | A | B | A | B | B |
| Ramachandran 2004 [60] | A | B | C | B | C |
| Yamanouchi 2005 [61] | A | A | A | A | A |
| Chen 2012 [62] | B | B | C | A | C |
| Jia 2005 [63] | B | A | A | A | B |
| Ling 2003 [64] | B | B | C | A | C |
| Liu 2013 [65] | A | B | C | B | C |
| Tang 2004 [66] | B | B | C | B | C |
| Wang 2011 [67] | A | A | C | B | B |
| Xu 2006 [68] | B | B | C | A | C |
| Yao 2008 [69] | A | A | A | A | A |
| Yao 2010 [70] | B | A | A | A | B |
| Zhang 2009 [71] | A | A | A | A | A |
| Goldstein 2003 [72] | A | A | A | B | B |
| Xuan 2012 [73] | B | B | C | B | C |
| Charpentier 2001 [74] | A | B | A | A | B |
| Formoso 2008 [75] | A | B | C | B | C |
| Ning 2006 [76] | B | B | C | B | C |
| Tessier 1999 [77] | B | B | C | A | C |
| Lawrence 2004 [78] | A | B | C | A | C |
| Hasegawa 2008 [79] | B | B | C | B | C |

**S3** Table. Continued

| **Study** | **Selection Bias** | | **Performance Bias** | **Attrition Bias** | **Overall Quality** |
| --- | --- | --- | --- | --- | --- |
|  | **Randomization** | **Allocation Concealment** | **Blinding** | **Dropout/loss to Follow-up** |  |
| Hu 2012 [80] | B | B | C | A | C |
| Li 2007 [81] | B | B | C | A | C |
| Li 2008 [82] | B | B | C | A | C |
| Rosenthal 2002 [83] | A | B | C | B | C |
| Salman 2001 [84] | B | B | C | B | C |
| Van de Laar 2004 [85] | A | A | A | B | B |
| Wang 1999 [86] | B | B | C | B | C |
| Wang 2007 [87] | B | B | C | A | C |
| Yang 2010 [88] | B | B | C | A | C |

In randomization procedure, A= adequate, B= inadequate or unknown; In allocation concealment, A= adequate, B= inadequate or unknown; In the use of blinding, A= adequate, B= mentioning of blinding but exact method unclear, C = non-blinded, inadequate or unknown; In dropout/loss to follow-up, A= overall dropout rate < 15%; B= overall dropout rate > 15%, or unknown. The overall quality of each study was classified as A= low risk of bias, high-quality trials; B= moderate risk of bias, moderate-quality trials; or C=high risk of bias, low-quality trials.
